# Combinatorial Binding in Human and Mouse Embryonic Stem Cells Identifies Conserved Enhancers Active in Early Embryonic Development

**DOI:** 10.1371/journal.pcbi.1002304

**Published:** 2011-12-22

**Authors:** Jonathan Göke, Marc Jung, Sarah Behrens, Lukas Chavez, Sean O'Keeffe, Bernd Timmermann, Hans Lehrach, James Adjaye, Martin Vingron

**Affiliations:** 1 Department of Computational Molecular Biology, Max-Planck Institute for Molecular Genetics, Berlin, Germany; 2 Department of Vertebrate Genomics, Max-Planck Institute for Molecular Genetics, Berlin, Germany; 3 Next Generation Sequencing Group, Max-Planck Institute for Molecular Genetics, Berlin, Germany; 4 The Stem Cell Unit, Department of Anatomy, College of Medicine, King Saud University, Riyadh, Saudi Arabia; MRC Laboratory of Molecular Biology, United Kingdom

## Abstract

Transcription factors are proteins that regulate gene expression by binding to cis-regulatory sequences such as promoters and enhancers. In embryonic stem (ES) cells, binding of the transcription factors OCT4, SOX2 and NANOG is essential to maintain the capacity of the cells to differentiate into any cell type of the developing embryo. It is known that transcription factors interact to regulate gene expression. In this study we show that combinatorial binding is strongly associated with co-localization of the transcriptional co-activator Mediator, H3K27ac and increased expression of nearby genes in embryonic stem cells. We observe that the same loci bound by Oct4, Nanog and Sox2 in ES cells frequently drive expression in early embryonic development. Comparison of mouse and human ES cells shows that less than 5% of individual binding events for OCT4, SOX2 and NANOG are shared between species. In contrast, about 15% of combinatorial binding events and even between 53% and 63% of combinatorial binding events at enhancers active in early development are conserved. Our analysis suggests that the combination of OCT4, SOX2 and NANOG binding is critical for transcription in ES cells and likely plays an important role for embryogenesis by binding at conserved early developmental enhancers. Our data suggests that the fast evolutionary rewiring of regulatory networks mainly affects individual binding events, whereas “gene regulatory hotspots” which are bound by multiple factors and active in multiple tissues throughout early development are under stronger evolutionary constraints.

## Author Summary

The mammalian body is composed of hundreds of distinct cell types. During embryogenesis, this diversity is created by multiple cell fate decisions and differentiation events. Embryonic stem (ES) cells provide the *in vitro* model to study differentiation and early development. Their pluripotent state is maintained by transcription factors such as OCT4, SOX2 and NANOG which bind to regulatory elements within the genome. Understanding the interplay between transcription factor binding, gene expression and cellular differentiation is key to understanding the development of the mammalian embryo. In this study we find that combinatorial binding of OCT4, SOX2 and NANOG in ES cells identifies enhancers which are associated with active transcription. We observe that these enhancers also frequently show activity at later developmental stages. Using data from mouse and human ES cells we find that these combinatorially bound enhancers which are active in pluripotent cells and development show extraordinarily high levels of binding conservation (>50%). Our analysis suggests that these conserved “gene regulatory hotspots” integrate the transcriptional network that promotes pluripotency into the gene regulatory networks that promote cell fate decisions and differentiation during early embryonic development.

## Introduction

Embryonic stem (ES) cells are derived from the inner cell mass of the blastocyst [1]. During the course of normal development, implantation of the blastocyst results in further differentiation into distinct cell types of the three primary germ layers that will later form the tissues and organs of the developing embryo [2]. This pluripotent state makes ES cells a unique *in vitro* cellular model system to study early embryogenesis. At the core of the regulatory network that maintains this state is a set of transcription factors amongst which OCT4 seems to play a key role [3], [4]. OCT4 has been shown to co-occupy many regulatory sites together with SOX2, NANOG and the co-activator p300 [5]. The potency of these transcription factors is demonstrated by their ability to induce pluripotency in mouse and human somatic cells. This was achieved by the ectopic expression of OCT4 and SOX2 together with either KLF4 and c-MYC, or NANOG and LIN28 [6], [7], [8].

The pivotal step in inducing and maintaining the pluripotent state occurs at the level of genomic DNA by the binding of transcription factors and co-factors that activate and repress gene expression. The largest fraction of the genome is non-coding with many non-coding elements being highly conserved. Even though it is expected that many of these elements harbor transcription factor binding sites and may act as enhancers, current understanding of the interplay between transcription factors and regulatory elements within the genome is limited. ChIP-Seq data pinpoints transcription factor binding sites not only in predefined regions such as promoters but in an unbiased way genome-wide. However, the high sensitivity comes along with a low specificity that makes identification of functional sites challenging. Nevertheless, in order to understand self renewal and pluripotency at the level of transcriptional regulation, it is crucial to identify a reliable set of regulatory elements that actively contribute to the regulation of gene expression in pluripotent cells such as embryonic stem cells and induced pluripotent stem cells.

ES cells reflect a very early time point of development. Many genes which are important for early embryogenesis have a conserved function in mouse and human. OCT4, SOX2 and NANOG for example are essential for maintaining the pluripotent state both in mouse and human ES cells [3], [9]. However, despite their conserved function, where these transcription factors bind seems to be highly species-specific: A comparison of mouse and human ES cells revealed that only about 5% of binding events of the key pluripotency factors OCT4 and NANOG are conserved at orthologous genomic locations in both species [10]. A study of genome-wide binding in liver tissue reported the same with only about 7% conserved binding events for the liver transcription factors CEBP and HNF4 between mouse and human [11]. These data show how fast cis-regulatory elements can evolve compared to coding sequence, yet we do not know what discriminates conserved from non-conserved binding events. Genome-wide comparisons give average values over all observed binding events. These numbers are influenced by many factors such as the choice of control, processing of the data, and p-value and false discovery rate cutoffs. The biological impact of individual binding events may therefore be very different, ranging from non-functional binding to binding events which are essential for the regulation of associated genes and the survival of the individual [12]. For that reason, the fraction of conserved binding events is currently unknown for a highly confident set of enhancers.

It is well known that transcription factors interact to regulate gene expression. It has been shown in mouse ES (mES) cells that combinatorial binding of Oct4, Sox2 and Nanog is associated with increased expression of nearby genes [5]. Here, we use combinatorial binding to increase the specificity of the ChIP-Seq technology in order to identify a highly confident set of regulatory elements. We investigate the association of these elements with the transcriptional co-activator Mediator, histone modifications and gene expression, to test whether the interactions of transcription factors provide a link between binding and activation of their target genes.

Studies in Drosophila suggest that the combination of binding sites plays an important role during the evolution of gene regulatory elements [13], however the effect of combinatorial binding on evolution in mammals is currently unknown. Analysis of mammalian genome sequences suggested that developmentally active enhancers are highly conserved [14]. Integration of genome-wide binding data from mouse and human ES and embryonal carcinoma (EC/NCCIT) cells and mouse developmental tissues allows us for the first time to study the evolution of gene regulation in the light of combinatorial binding and developmental activity in mammalian cells using an *in vitro* system. Our analysis indicates that both characteristics contribute to the evolutionary constraint on regulatory elements and suggests that the integrated data represents an essential set of conserved enhancers that links pluripotency with early embryonal development.

## Results

**Figure 1:**
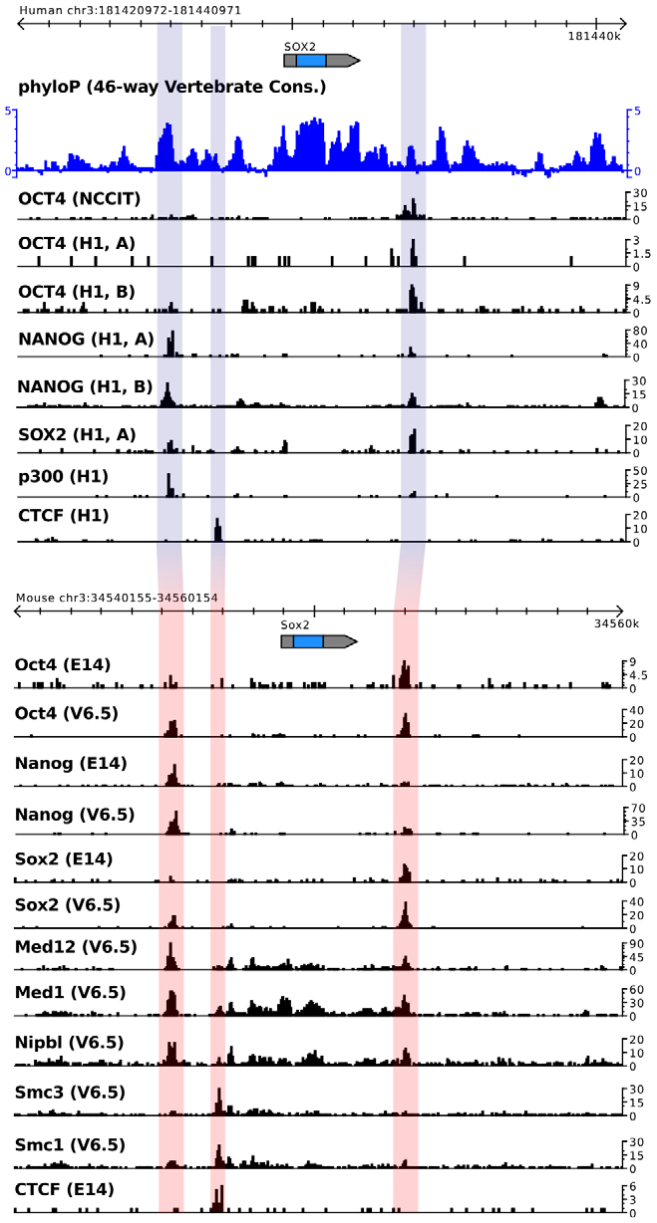
Overview of genome-wide binding data in human and mouse embryonic stem cells and embryonal carcinoma cells. Shown is the locus of the *SOX2* gene in the human genome (top), along with mapped reads for OCT4, SOX2, NANOG and p300. Individual experiments are shown separately. The orthologous locus in the mouse genome is aligned at the bottom along with mapped reads from the individual experiments.

We mapped genome-wide binding data of Oct4, Sox2 and Nanog in mES cells to study the effect of combinatorial binding. We further mapped binding data from the transcriptional co-activators p300 and Mediator subunits Med1 and Med12 which are important to activate gene expression by linking regulatory elements with the basal transcriptional machinery. We additionally mapped binding data from Cohesin (subunits Smc1 and Smc3) and CTCF which are involved in gene regulation through DNA loop formation [15]. Using MACS [16] we identified sets of potential binding events (ChIP-Seq “peaks”) for every factor. We discarded all peaks with a p-value>1e-05 and peaks that were detected in the control data (“full data set”). As a control for the influence of the p-value cutoff, we additionally analyzed the data using only the top 10% of peaks (sorted by p-value) from every experiment (“stringent data set”). We intentionally did not choose a false discovery rate (FDR) cutoff, since the FDR (as estimated by MACS) is heavily dependent on the control data [16] which is lacking for some experiments (see supplementary Figures S1). To compare genome-wide binding in mouse and human ES cells we processed data from human cells in the same way (see supplementary Table S1 for a complete listing of accession numbers, mapped reads and number of peaks). Important insights have been obtained from studies using ChIP-on-chip data [3], [17], however due to its limitation to promoter regions we did not integrate this data into our analysis. The complete data used in this study is available at the European Nucleotide Archive (supplementary Table S1) and can be accessed at http://enhancer.molgen.mpg.de, where we provide a human and mouse genome browser displaying genome-wide binding profiles, major histone modifications and RNA-seq data [18]. Figure 1 shows the aligned *SOX2* locus in the mouse and human genomes along with the data used in this study.

### Combinatorial binding detects functional interactions

**Figure 2:**
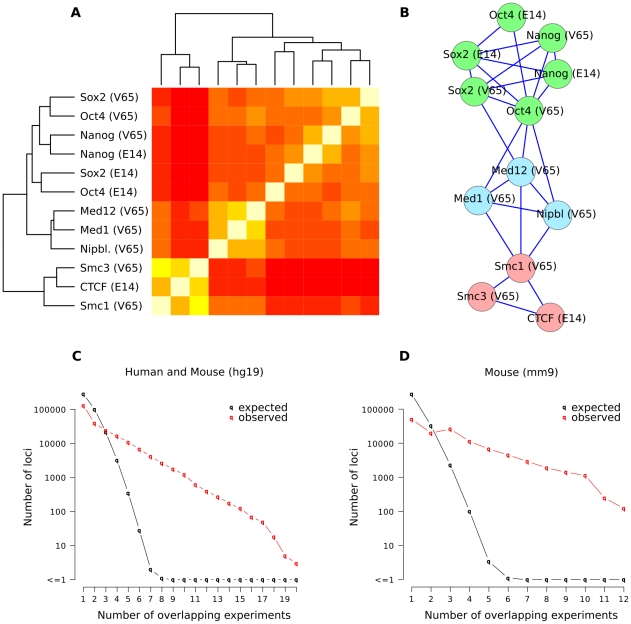
Co-localization within the genome identifies known protein interaction. (**A**) Clustering of genome-wide binding profiles from mES cells based on the number of shared binding events identifies three main classes: Enhancer binding (Oct4, Sox2, Nanog), Insulator binding (CTCF, Smc1, Smc3) and Mediator associated binding (Med1, Med12, Nipbl). (**B**) Protein interaction network inferred from genome-wide binding data. Edges represent the pairwise similarities with a z-score above a threshold. (**C–D**) The number of overlapping experiments is much higher than expected by chance, both for mouse binding data (mm9, D) and human binding data together with the aligned mouse data (hg19, C). Randomized data sets show only very few cases where more than five experiments overlap (black line). The data used in this study shows a much stronger overlap with many loci where binding was detected in more than five experiments (red line).

As a first step toward analyzing combinatorial binding we calculated the similarity of the genome-wide binding profiles for all factors (Figure 2A–B, see Materials and Methods). These similarities identify three distinct clusters: Enhancer binding (Oct4, Nanog, Sox2), Insulator binding (CTCF, Cohesin subunits Smc1 and Smc3a) and transcriptional co-activation (Mediator subunits Med1 and Med12). Interestingly, pairwise distances from genome-wide data on DNA-protein interactions reproduce known protein-protein interactions [19] (Figure 2B): CTCF interacts with Cohesin at insulator elements, Oct4, Sox2 and Nanog interact at enhancers, and Mediator plays a central role by integrating signals from distant regulatory elements and Cohesin. To test whether the amount of overlap of transcription factor binding that we observed can be expected by chance, we calculated the overlap of position-randomized binding events. Overall, the overlap observed in the data is much higher than expected by chance (Figure 2C–D). These results support the notion that the combination of binding events reflects functional interactions between the proteins themselves.

### The Mediator complex co-localizes at combinatorially bound loci

**Figure 3:**
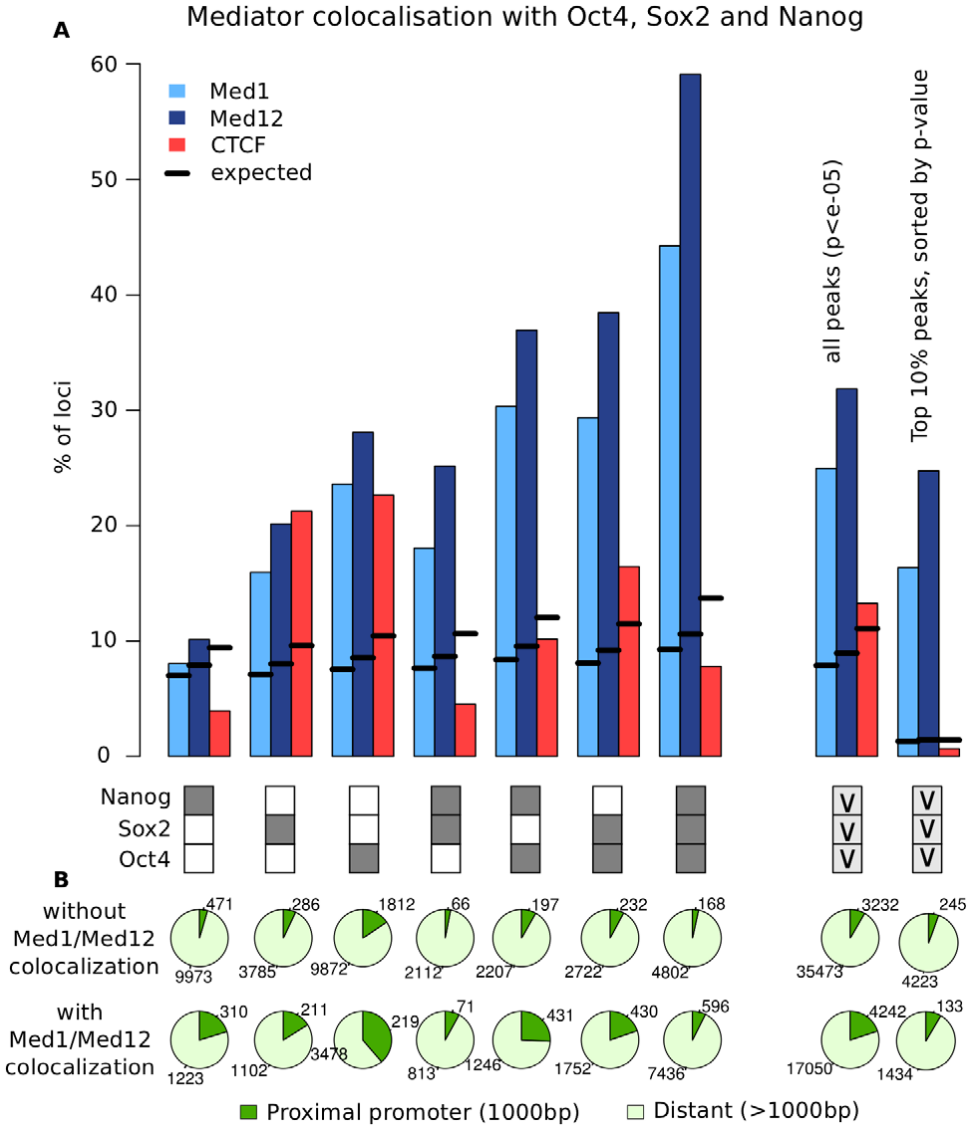
Mediator co-localizes with Oct4, Sox2 and Nanog at combinatorially bound enhancers. (**A**) Bars indicate the fraction of loci where Med1, Med12 and CTCF binding can be observed, depending on the combination of Oct4, Sox2 and Nanog, indicated by boxes below. Dark boxes indicate binding, white boxes indicate no binding (“AND” relation), light grey boxes with “v” indicate binding of at least one factor (“OR” relation). Both Med1 and Med12 preferentially co-localize at loci bound by Oct4, Sox2 and Nanog simultaneously. Combinatorial binding is more sensitive than a stringent control of false positives: The 10% most significant peaks are significantly associated with Med1 and Med12, however, the overall fraction is much lower compared to combinatorially bound loci. CTCF serves as a control to estimate unspecific binding. (**B**) The majority of loci bound by Oct4, Sox2 and Nanog are more than 1000 bp away from the nearest transcription start sites for all possible combinations (indicated by boxes above). Mediator co-localization mainly occurs at distant regulatory sites, showing that the increased overlap of Med1/Med12 at combinatorially bound loci is not caused by promoter specific co-localization.

Combinatorial binding of Oct4, Sox2 and Nanog in mES cells has been reported to influence gene expression [5], however the exact mechanism is unclear. Since the Mediator complex has a central role in linking enhancers with activation of gene expression [15], [20], [21], we examined whether the binding combination of Oct4, Sox2 and Nanog influences co-localization of the Mediator subunits Med1 and Med12 at enhancers. For all possible combinations we calculated the fraction of loci where Med1 or Med12 co-localizes (Figure 3A). Since these loci vary by number and size, we calculated the expected overlap from randomized data sets (Hyper geometric test, see Materials and Methods). Between 5% and 30% of loci bound by Oct4, Nanog or Sox2 individually co-localize with Med1 or Med12 (Figure 3A). In contrast, loci bound by Oct4, Nanog and Sox2 simultaneously (further referred to as combinatorially bound loci) co-localize much more frequently with Med1 (44%, z-score 155.9) and Med12 (59%, z-score 215.5).

Co-localization of DNA binding proteins could be unspecific, for example due to binding at open chromatin regions (see [12] for a review). Unspecific co-localization is not accounted for with the theoretical expected overlap. To control for this effect we need to calculate co-localization of factors which we know are unrelated. Figure 2 shows that CTCF largely binds to different regions than the enhancer binding proteins Oct4, Sox2 and Nanog [22], therefore CTCF co-localization should be depleted at combinatorially bound loci. We observe that CTCF overlaps with individual binding events of Sox2 and Oct4. In contrast, CTCF co-localization is significantly depleted at loci bound by Oct4, Sox2 and Nanog simultaneously (z = −10.5). This suggests that combinatorial binding reduces unspecific co-localization and confirms the association of the Mediator complex with combinatorial bound loci.

The strength of the ChIP-Seq signal (“binding intensity”) is likely to hint at important binding sites. We calculated the association of Mediator with Oct4, Sox2 and Nanog independent of their combination for a data set that only includes the top 10% peaks (sorted by p-value) for every experiment. In this set, CTCF co-localization is depleted showing that a stringent p-value cutoff efficiently reduces unspecific overlap (Figure 3A). However, combinatorial binding is a much more sensitive indicator for Mediator co-localization, as only 16% of Oct4, Sox2 and Nanog loci show Med1 co-localization when we look into high intensity peaks (25% with Med12), compared to 44% for the combinatorially bound loci in the full data set (59% for Med12). The binding combination has a similar influence in both the stringent and the full data set (supplementary Figure S2), re-assuring us that the particular choice of p-value cutoff is of little importance in this analysis.

Since Mediator occupies many promoters in the genome, transcription factors that bind preferentially to promoter regions would be expected to show co-localization with Med1 or Med12. To test whether the interaction between Oct4, Sox2 and Nanog occurs mainly at the promoter thereby causing the observed Mediator co-localization, we calculated the fraction of promoter and enhancer bound loci for all binding combinations. The majority of loci bound by Oct4, Sox2 and Nanog are at distant regulatory elements (61%–97%), even when Mediator co-localization can be observed (Figure 3B). This shows that the increased overlap at combinatorially bound loci reflects specific binding at distant regulatory elements and is not caused by simultaneous occupation of the proximal promoter of actively transcribed genes.

**Figure 4:**
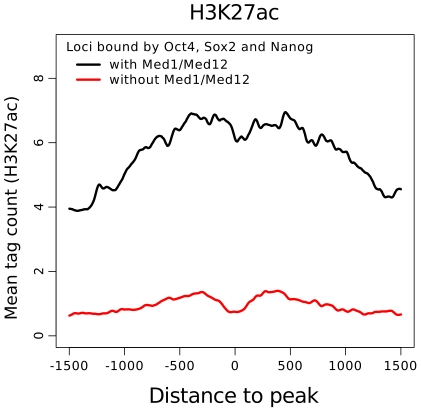
Average H3K27ac ChIP-Seq signal in mES cells around combinatorially bound loci. Loci bound by Oct4, Sox2 and Nanog together with Mediator are enriched in H3K27ac, a mark associated with active enhancers (black line). In contrast, loci without Mediator co-localization show a much weaker enrichment (red line) suggesting that Mediator associates with active enhancers.

**Figure 5:**
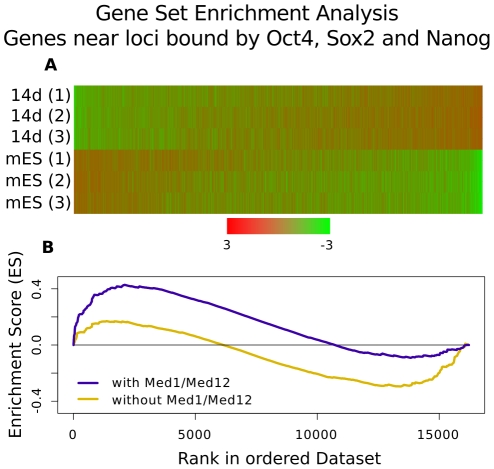
Gene Set Enrichment Analysis (GSEA) of genes near combinatorial binding events. (**A**) Expression of genes in mES cells (V6.5) and differentiated cells after 14 days (14d), sorted by the signal-to-noise ratio obtained from the GSEA software [24]. (**B**) The random walk that describes the gene set enrichment over genes sorted by their rank according to signal-to-noise ratio (similar sorting as in (A)). Set 1 (Oct4, Sox2, Nanog and Med1/Med12 in blue) is enriched in genes active in mES cells (enrichment score 0.43, p-value = 0.0), set 2 (Oct4, Sox2, Nanog without Med1/Med12 in yellow) is enriched in genes active in differentiated cells (enrichment score −0.3, p-value = 0.05). Combinatorial binding of Oct4, Sox2 and Nanog identifies active and poised enhancers; Mediator is associated with active gene expression.

The strong association of combinatorial binding with Mediator suggests that Mediator bound loci are functionally different from loci without Mediator binding. We used histone modification profiles and gene expression of nearby genes to test for functional differences. Combinatorially bound loci occupied by Mediator are strongly enriched in H3K27ac, a mark for active enhancers, compared to loci without Mediator co-localization (Figure 4) [23]. To test the effect of Mediator binding on gene expression, we performed a gene-set enrichment analysis (GSEA) [24] using expression data from mES cells compared to differentiated cells after 14 days [25]. We sorted all genes according to their difference between ES cells and differentiated cells (Figure 5A) and then calculated the enrichment score for genes near loci bound by Oct4, Sox2 and Nanog with Med1/Med12 (group 1) and without Med1/Med12 (group 2). Group 1 shows a significant enrichment in genes expressed in stem cells (Enrichment scores 0.43, p-value 0.0) (Figure 5B). Interestingly, group 2 shows a stronger enrichment in genes which are expressed in differentiated cells (Figure 5B, enrichment score −0.3, p = 0.05), suggesting that Oct4, Nanog and Sox2 might co-occupy poised enhancers. Both histone profiles and gene expression data support the notion that combinatorial binding identifies enhancers in embryonic stem cells while Mediator co-localization determines their activity.

### Combinatorial binding identifies developmental enhancers in embryonic stem cells

Binding of Oct4, Sox2 and Nanog frequently occurs near developmental genes [3] and gene expression data suggests that genes near combinatorial binding events are indeed up-regulated after differentiation. The majority of loci bound by Oct4, Sox2 and Nanog together with Med1 or Med12 are likely to act as enhancers in embryonic stem cells. However, the function of loci near inactive genes is unclear. Since many of these genes are active during development, we tested whether Oct4, Sox2 and Nanog co-occupied loci act as early developmental enhancers. We used a set of tissue-specific enhancers obtained from mouse embryos at day e11.5 [14], [26], a stage where neither Oct4 nor Nanog is expressed. Nevertheless, 9% (z = 27.5) of combinatorially bound loci in ES cells overlap with developmental enhancers. Enhancers that are active in development show an enrichment of H3K27ac in neuronal progenitor cells (supplementary Figure S3), supporting that these indeed become active after differentiation. It is likely that some of these regulatory elements are in a poised state. Poised enhancers have been identified in embryonic stem cells by unique chromatin modification signatures [23], [27], however, this is the first evidence for active participation of Oct4, Sox2 and Nanog in this poised state. Our analysis suggests that binding of pluripotency-associated transcription factors at developmental enhancers might be a key feature of the ability of pluripotent stem cells to differentiate into distinct cell types. Usage of such “shared enhancers”, which are active in multiple stages during differentiation, links the gene regulatory networks of embryonic stem cells with the networks for early development at the level of transcriptional regulation.

### Combinatorial binding events are conserved in evolution

Using data from human ES cells, we investigated whether combinatorial binding can further help in discriminating conserved and non-conserved binding events. To test this, we first mapped genome-wide binding data for OCT4, SOX2 and NANOG from human ES cells and OCT4 from human embryonal carcinoma (EC) cells using the same procedure as described above (supplementary Table S1 for Accession numbers, mapped reads and number of peaks). EC cells are the malignant counterpart of ES cells [28], however they possess a distinct set of binding sites, extending the repertoire of potential OCT4 bound loci. We used whole-genome alignments to assign binding events in mES cells to their orthologous loci in the human genome, retaining only those that could be aligned uniquely [29]. We call a binding event “conserved” if binding of the same factor can be observed at the aligned loci in the human and mouse genome.

**Figure 6:**
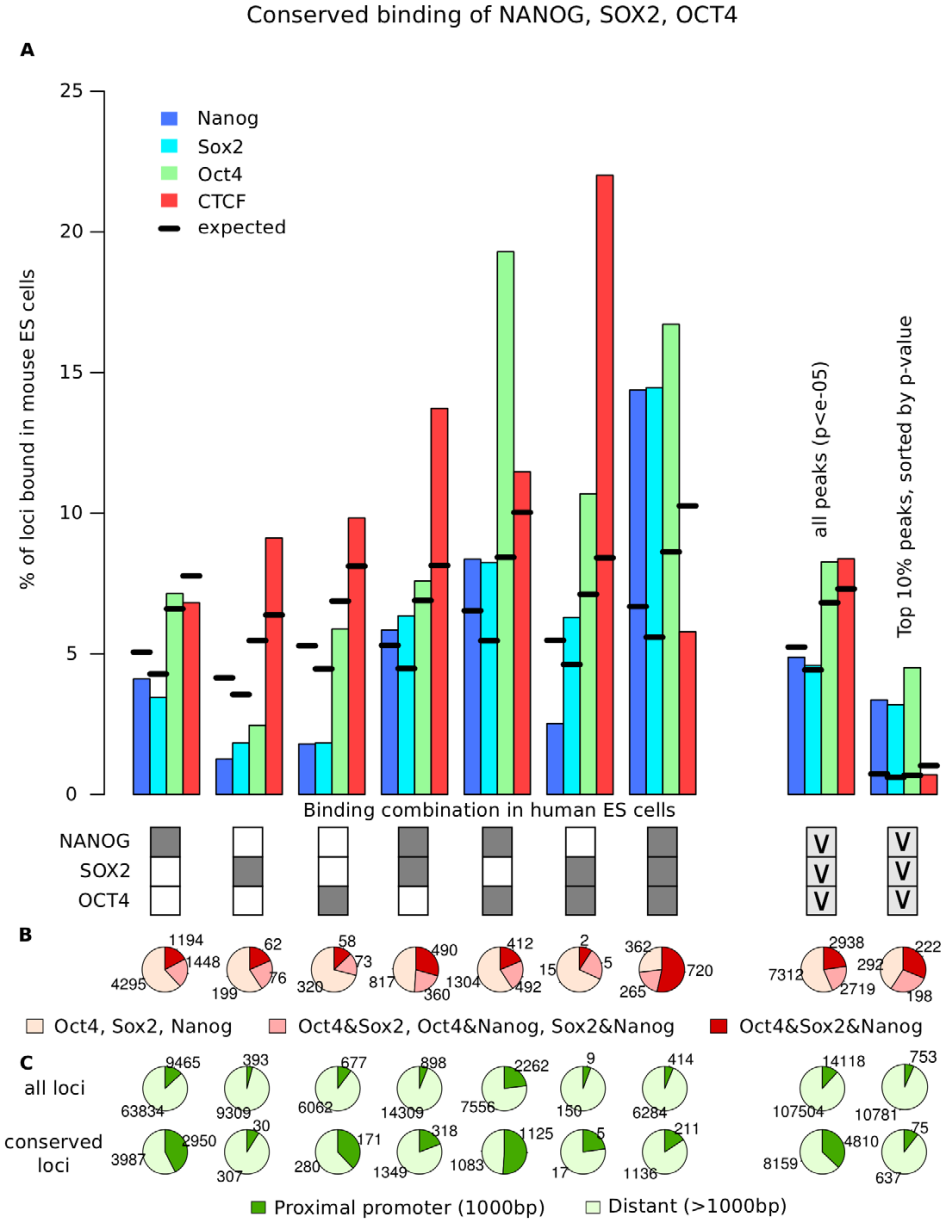
The combination of OCT4, SOX2 and NANOG influences conservation of binding events. (**A**) Bars indicate the fraction of loci where binding of Nanog, Sox2, Oct4 or CTCF can be observed at the orthologous locus in mouse ES cells for all combinations of OCT4, SOX2 and NANOG in human ES cells as indicated by the boxes below. Dark boxes indicate binding, white boxes indicate no binding (“AND” relation), light grey boxes with “v” indicate binding of at least one factor (“OR” relation). Combinatorial binding of OCT4, SOX2 and NANOG shows the largest fraction of conserved binding for Oct4, Sox2 and Nanog in mouse. Again, combinatorial binding is more sensitive than a stringent control of false positives, as is estimated by conservation at 10% most significant peaks. (**B**) The fractions of binding combinations in mES cells at conserved loci (for all combinations of binding in human cells as indicated by the boxes above). Combinatorial binding of Oct4, Sox2 and Nanog in mES cells is much higher at combinatorially bound loci in human, suggesting that combinatorial binding is conserved in evolution. (**C**) The fraction of proximal and distant binding sites for conserved and non-conserved binding events, split up according to the combinations of binding as indicated by the boxes above. The majority of conserved binding events are distant regulatory elements. Conserved binding events are more frequently in the proximal promoter than non-conserved binding events.

For every combination of OCT4, SOX2 and NANOG binding we calculated the fraction of conserved binding events for every factor (Figure 6A). Indeed, combinatorial binding is a good predictor for conservation: Less than 5% of individual binding events are conserved, which is less than expected. In contrast, about 15% of binding events at loci which are simultaneously co-occupied by OCT4, SOX2 and NANOG in hES cells show conserved binding of the respective transcription factor in mES cells (z-scores = 33.6, 41.3, 31.1, Figure 6A). We additionally calculated the number of transcription factors that bind at conserved loci in mouse for all combinations of OCT4, SOX2 and NANOG in human cells (Figure 6B). 53% of combinatorial binding events in human are simultaneously occupied by Oct4, Sox2 and Nanog in mouse, showing that combinatorial binding is likely to be a conserved property of regulatory elements in ES cells.

Since CTCF does not show significant association with combinatorially bound loci in mouse (Figure 2, 3), we use CTCF binding in mES cells to estimate unspecific conservation. CTCF binding is significantly depleted at combinatorially bound loci (Figure 6A, z-score = −6.8), showing that the increased conservation is specific to the combination of transcription factors. In contrast, CTCF binding can be observed at higher levels for all other binding combinations, most prominently OCT4 with SOX2. The combination of OCT4 and SOX2 without NANOG scarcely occurs genome-wide (159). In contrast, the combination OCT4, SOX2 and NANOG occurs 6698 times. The high levels of CTCF at OCT4/SOX2 loci is therefore likely to be unspecific and of low relevance. We further tested the enrichment of CTCF using the stringent data set that only contains the 10% most significant peaks (supplementary Figure S4). In this stringent data set, CTCF levels drop below 5% for all combinations. This suggests that CTCF enrichment is an artifact of the large data set (false positive binding events). To see if a stringent p-value can be used to obtain similar estimates, we calculated the conservation independently of the binding combinations for the full and stringent data set (Figure 6A). Less than 5% of binding events are conserved between mouse and human in the stringent data set. This is higher than expected. However, combinatorial binding is a more sensitive indicator for conservation (3–5% conserved events for p-value cutoff vs. 14–17% for combinatorial binding), probably because many true binding sites will be lost in the stringent data set.

Interestingly, the fraction of loci within the proximal promoter (+−1000 bp) is higher for conserved binding events compared to non-conserved binding (Figure 6C), thus suggesting that the promoter is under stronger evolutionary constraint. However, the majority of binding events are distant from the predicted transcription start sites. The increased level of conservation at combinatorially bound loci is therefore not caused by a bias towards promoter binding.

### OCT4, SOX2 and NANOG binding is highly conserved at developmental enhancers

**Figure 7:**
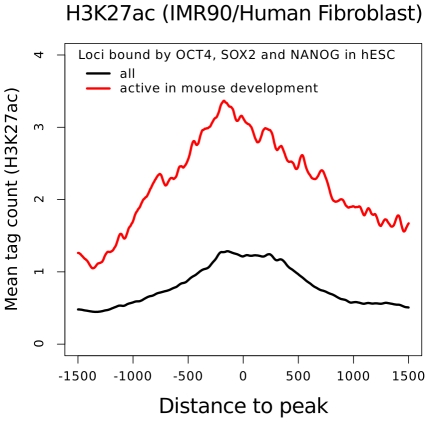
Average Fibroblast ChIP-Seq signal profile around loci bound by OCT4, SOX2 or NANOG in hES cells. Enhancers bound by OCT4, SOX2 or NANOG which are active in mouse development (red line) are enriched in H3K27ac in human fibroblast cells (IMR90) supporting that many of these enhancers are developmentally active in human as well.

Studies in mouse have revealed that developmental enhancers often show high sequence conservation [14], [30]. However, sequence conservation alone is very limited in its ability to estimate *in vivo* binding conservation [11]. Here, we observe that 26% of combinatorially bound loci which are conserved between mouse and human ES cells are developmental enhancers in the mouse (supplementary Figure S5). This suggests that many enhancers bound by OCT4, SOX2 and NANOG are developmental enhancers in human. Interestingly, these enhancers show increased levels of H3K27ac in human fibroblast cells (Figure 7), suggesting that many combinatorially bound enhancers in embryonic stem cells indeed become active after differentiation. This finding enables us for the first time to study *in vitro* binding conservation at developmental enhancers between mouse and human.

**Figure 8:**
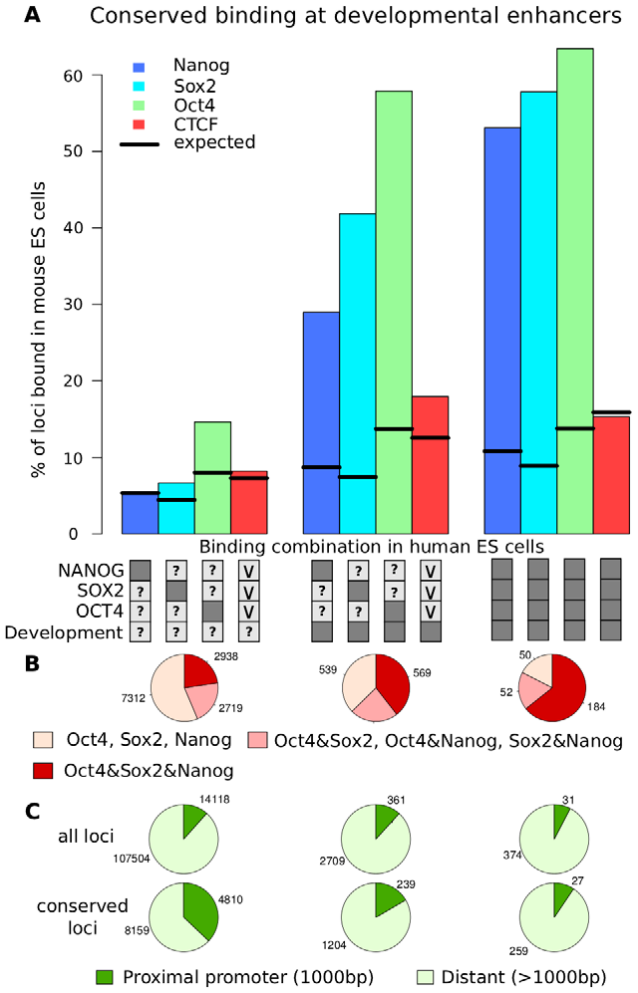
Binding conservation in embryonic stem cells is increased at developmental enhancers. (**A**) Bars indicate the fraction of loci where binding of Nanog, Sox2, Oct4 and CTCF can be observed at the orthologous locus in mouse ES cells for all combinations of OCT4, SOX2 and NANOG in human ES cells discriminated by developmental activity as indicated by the boxes below. Dark boxes indicate “AND” relation, light grey boxes with “v” indicate “OR” relation, “?” indicates no restriction. Combinatorial binding events at developmentally active enhancers show the highest levels of binding conservation between mouse and human ES cells (>50%). (**B**) The fractions of binding combinations in mES cells at conserved loci (for all combinations indicated by the boxes above). The majority of conserved binding events at developmentally active enhancers where OCT4, SOX2 and NANOG bind simultaneously show combinatorial binding of Oct4, Sox2 and Nanog in mouse ES cells. (**C**) The fraction of proximal and distant binding sites for conserved and non-conserved binding events (split up according to the combinations of binding as indicated by the boxes above). The majority of conserved binding events are distant regulatory elements.

We calculated the fraction of conserved binding events for the different combination of transcription factors, this time discriminating binding events by developmental activity (Figure 8). Strikingly, 63%, 58% and 53% of OCT4, SOX2 and NANOG binding events are conserved in mouse at enhancers that are active in early development (Figure 8A). This number is drastically higher than previous estimations [10] and shows that combinatorial binding together with developmental activity of the bound loci are strong indicators for binding conservation in embryonic stem cells.

**Figure 9:**
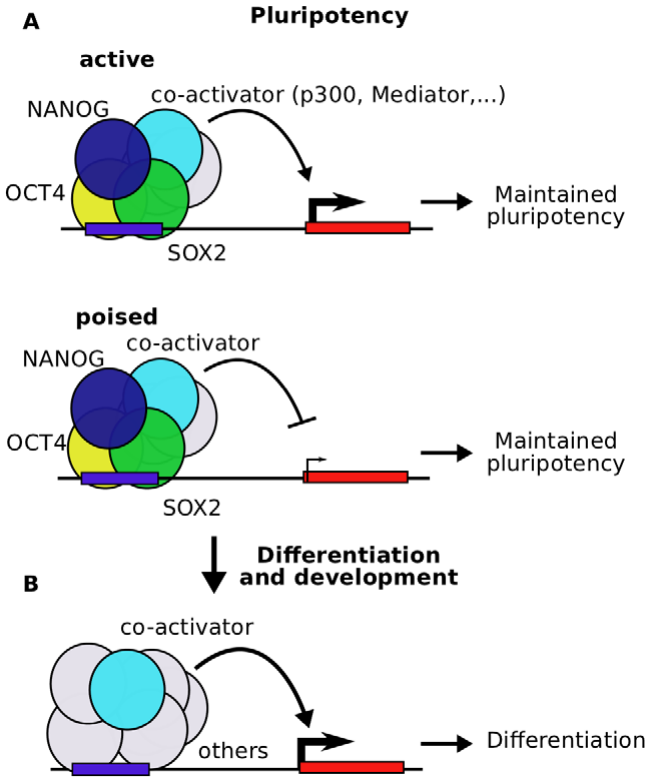
Model for “gene regulatory hotspots”. (**A**) Enhancers are bound by OCT4, SOX2 and NANOG together with p300 in embryonic stem cells. These enhancers maintain pluripotency by activating gene expression in ES cells (top) or poisoning expression for activation after differentiation (bottom) (**B**) After differentiation of the cell, the same enhancers are bound by p300 in developmental tissues together with other transcription factors. The target gene is expressed. We propose that enhancers which recruit multiple transcription factors in different stages of development are gene regulatory hotspots which are crucial to connect the regulatory networks of pluripotency and development. These enhancers show higher sequence conservation compared to individual, isolated binding events which active in single cell types.

The prominent difference in conservation between individual, isolated binding events and combinatorial binding events at enhancers which are active in multiple cell types would suggest the existence of “gene regulatory hotspots” which are highly conserved in evolution (Figure 9). These hotspots are enhancers which recruit multiple, interacting transcription factors in pluripotent cells where they can be in an active or poised state. The very same element recruits different sets of transcription factors after differentiation and during development. This complex regulatory activity might lead to the high level of conservation that we can observe in this study and discriminates these hotspots from isolated transcription factor binding sites.

The element downstream of *SOX21* is such an example and illustrates the intimate connection between embryonic stem cells, pluripotency and development (supplementary Figure S6). SOX21 plays a pivotal role during brain development by promoting neuronal differentiation [31]. The downstream regulatory element is ultra-conserved with high sequence similarity in human, mouse and zebrafish, where it is always in close proximity to the *SOX21* gene. The cis-regulatory element is bound by OCT4, SOX2, NANOG and p300 in human ES cells and Oct4, Sox2, Nanog and p300 in mouse ES cells (supplementary Figure S6A–B). During mouse midbrain and forebrain development, this element is bound by p300 and expression data shows that Sox21 is indeed over expressed in forebrain compared to the whole embryo at day 11.5 [14]. The human element was tested *in vivo* in mouse and showed reproducible activity in forebrain, midbrain, hindbrain and neural tube (supplementary Figure S6C) [32]. The same element is conserved in amphioxus where it is associated with the *SOX21* ortholog *soxB2*. The amphioxus sequence was tested in zebrafish where it showed reproducible activity in forebrain [33] (supplementary Figure S6D). The conserved enhancer downstream of *SOX21* is therefore a unique example of a functionally and genetically ultra-conserved cis-regulatory element that is bound in ES cells and active during development. This finding is indeed remarkable as it has been estimated that amphioxus split from vertebrates about 550 million years ago [34].

### Conserved binding events are associated with genes involved in transcriptional regulation and early development

The outcome of transcription factor binding events is ultimately determined by the function of the genes that they regulate. We found 720 conserved loci bound by OCT4, SOX2 and NANOG in human and mouse ES cells, associated with 608 genes nearby. Amongst these putative target genes are *OCT4*, *SOX2*, *LEFTY1*, *JARID2* and many other well known factors associated with pluripotency. A number of genes also show strong species-specific binding patterns, most prominently *Esrrb*, an interaction partner of Oct4 [35] which is almost exclusively bound in mouse ES cells (supplementary Figure S7).

To obtain a more general picture of the downstream target genes of conserved combinatorial binding events we performed a Gene Ontology (GO) enrichment analysis using the Genomic Regions Enrichment of Annotations Tool [36]. We selected all combinatorially bound regions as background. The subset of conserved combinatorially bound loci is significantly enriched in the terms pattern specification process (p = 4.7e-13), regionalization (2.5e-12) and developmental induction (8.4e-8). Even though the background set is already enriched in developmental GO terms (amongst others developmental induction, p-value 2e-8), the association of conserved combinatorial binding events with developmental processes is even stronger. In support of this, genes such as SOX21 [37], FGF4, NEUROG3 [38] and CDX2 [39] which are located near conserved combinatorial binding events have been shown to be important for directing differentiation of ES cells. This implies that conserved combinatorial binding events in ES cells have extensive downstream impact by controlling key regulatory genes involved in differentiation and provides further evidence for the tight connection between pluripotency and early development mediated by OCT4, SOX2 and NANOG through binding at developmental enhancers.

## Discussion

The enormous amount of genome-wide binding data produced in recent years has improved our understanding of the self-renewing and pluripotent state of embryonic stem cells [3], [5], [27], [40]. By integrating data from ES cells with developmental enhancers we discovered that the very same regulatory elements bound by key pluripotency factors in ES cells frequently act as enhancers during early development. This finding provides an unknown link between the gene regulatory networks of ES cells and early development at the level of transcriptional regulation.

The finding that binding at these developmental enhancers is highly conserved in mouse and human ES cells suggests that these elements are crucial for the maintenance of the pluripotent state. Nevertheless, some questions remain. Based on our data we cannot explain why in pluripotent cells OCT4, SOX2 and NANOG bind to enhancers which are also active at developmental stages when neither NANOG nor OCT4 are expressed. It is likely that these elements are poised for activation [23], [27], and an open chromatin state might be maintained throughout development to enable recruitment of transcription factors, co-activators, or histone modification proteins throughout cellular specification. These enhancers bound in multiple developmental stages by multiple factors show properties of gene regulatory hotspots, elements that influence gene expression in numerous cell types from pluripotent cells to at least cells of the mouse embryo at day 11.5. The existence of such gene regulatory hotspots could explain the extraordinarily high level of binding conservation observed in ES cells, since mutations of these elements would influence a major part of early embryogenesis. In contrast to these hotspots, loss of individual binding events can more easily be substituted by nearby binding events, and is likely to influence only a limited number of cell types. Our analysis suggests that the fast evolutionary rewiring of regulatory networks indeed mainly affects individual binding events, while combinatorial binding at these regulatory hotspots is under stronger evolutionary constraint.

The definition of combinatorial binding in this study relies on ChIP-Seq technology. We define combinatorial bound loci as genomic regions were we observe binding of different transcription factors in similar cell types. These experiments are independent of each other and reflect measures from a mixed population of cells. Co-localization could therefore be observed without direct physical interactions (for example through indirect interaction or competitive binding). However, based on the results of this and other studies [35], [41] we believe that co-localization as observed by the ChIP-Seq technology indeed reflects combinatorial binding.

One of the difficulties in analyzing genome-wide datasets is how to discriminate true binding sites from false positive binding sites. It is impossible to identify a set of exclusively true binding sites, due to technical limitations, but also due to biological variation since many binding events will only be important under specified developmental cues. A more stringent p-value cutoff decreases the fraction of false binding sites in the data while at the same time true positive binding events will be lost. Combinatorial binding is likely to select for true binding sites as well, since non-functional binding events are unlikely to be detected in multiple experiments (supplementary Figure S10). However, combinatorial binding is different from a stringent control of false positives as can be seen by Mediator co-localization and binding conservation (Figures 3 and 6). It has been shown that groups of transcription factor binding sites are more likely to be conserved than isolated sites [42] which supports the value of combinatorial binding for transcriptional regulation. This is an important insight for future studies, which should consider the combination of transcription factors for defining regulatory networks.

One limitation of the ChIP-Seq technology is that we cannot exclude combinatorial binding at isolated binding events. Weak or sporadic combinatorial binding events might be missed and therefore wrongly assigned as individual binding events (false negatives). We compared the results using two different cutoffs, a loose cutoff (full data set) with few false negatives and a very stringent cutoff (stringent data set) with many false negatives. The results largely agree: Combinatorial binding events (OCT4, SOX2, NANOG) consistently show the strongest association with Mediator (supplementary Figure S2) and highest levels of binding conservation (supplementary Figures S4 and S9). This suggests that the influence of the p-value cutoff and false negative binding events is limited on this analysis.

Most of the binding data in this study is obtained from embryonic stem cells. We integrated data sets from two mouse cell lines (V6.5 and E14). Interestingly, loci bound in both cell lines are much more likely to show co-localization with Mediator (supplementary Figure S8A). In human, we extended the available data by OCT4 ChIP-Seq from embryonal carcinoma cells to obtain data from different cell lines. We observe that, loci bound in EC and ES cells are much more likely to show combinatorial binding (supplementary Figure S8B). Therefore employing closely related cell lines is a biologically relevant approach for identifying important binding sites when data on combinatorial binding is not available.

Developmental cues that lead to differentiation of cells during early embryogenesis involves binding of transcription factors at regulatory sequences in the genome. We have demonstrated that in ES cells, the combination of transcription factors that bind to regulatory elements is important for transcriptional activation. Combinatorial binding of OCT4, SOX2 and NANOG identifies enhancers characterized by H3K27ac and Mediator co-localization. Many of these combinatorially bound enhancers are active during early development. The comparison of mouse and human ES cells shows that both combinatorial binding and multiple activity of enhancers in ES cells and development increase the evolutionary constraint. The set of conserved combinatorially bound embryonic stem cell enhancers is available (supplementary Table S2) and might be helpful as a set of putative human developmental enhancers. Our analysis suggests that the fast evolutionary rewiring of regulatory networks mainly affects individual binding events. In contrast to these events, there is a group of conserved enhancers in the genome which recruit multiple interacting factors and are active in multiple tissues of the developing embryo (Figure 9). Many of these “gene regulatory hotspots” are under strong evolutionary constraints and seem to play a major role by linking the regulatory networks of cellular differentiation during early mammalian development.

## Materials and Methods

### Cell culture

NCCIT cells were grown in high-glucose DMEM supplemented with 10% FCS (Biochrom, Berlin/Germany), 2 mM glutamine, and penicillin/streptomycin on conventional tissue culture dishes.

### Chromatin immunoprecipitation

Human NCCIT cells were grown to a final count of 5×10^7^–10^8^ cells for each Immunoprecipitation. Cells were chemically crosslinked by the addition of one-tenth volume of fresh 11% formaldehyde solution for 10 minutes at room temperature. Cells were rinsed twice with 1×PBS and harvested using a silicon scraper and flash frozen in liquid nitrogen and stored at −80°C prior to use. Cells were resuspended, subjected to lysis buffers, and sonicated to solubilize and shear crosslinked DNA. Sonication conditions vary depending on cells, culture conditions, crosslinking, and equipment. We used a BRANSON 250 and sonicated at power 3 for 11:00 min with 30% Duty Cycle at 4°C while samples were immersed in an ice bath. The resulting whole-cell extract (WCE) was incubated overnight at 4°C with 100 µl of Dynal Protein G magnetic beads that had been pre-incubated with either 10 µg of OCT4 antibody or a non-specific control antibody (normal goat IgG, sc-2028).

Beads were washed five times with RIPA buffer and once with TE containing 50 mM NaCl. Bound complexes were eluted from the beads by heating at 65°C with occasional vortexing, and crosslinking was reversed by overnight incubation at 65°C. Whole-cell extract DNA (reserved from the sonication step) was also treated for crosslink reversal. Immunoprecipitated DNA and whole-cell extract DNA were then purified by treatment with RNase A, proteinase K, multiple phenol∶chloroform∶isoamyl alcohol extractions and precipitation with ethanol. Purified DNA was amplified using a one-stage random PCR protocol [43].

### Library preparation

Input and ChIP-Seq material was purified using QIAquick spin columns and buffer QG (Qiagen) according to the manufacturer's protocol. 200 µg of fragmented DNA were subjected to single end library preparation using the genomic DNA sample prep kit (#FC-102-1002, Illumina) according to the manufacturer's instructions with the following modifications. End repair was performed in the presence of 0,25 mM dNTPs Mix in a total volume of 100 µl, A-tailing was performed in a total volume of 50 µl. Adapters were ligated to the DNA fragments using 10 µl of ‘Adapter oligo mix’ and in a total reaction volume of 50 µl. Libraries were size selected on a 2% agarose gel for fragments of 150–250 bp. After size selection, PCR comprising of 17 amplification cycles was carried out following the instructions manual. Libraries were quantified on a Qubit fluorometer using the QuantIt dsHS Assay Kit (Invitrogen).

### Illumina Genome Analyzer sequencing

After library quantification a 10 nmol stock solution of the amplified library was created. 4 pM of the stock solution were loaded onto the channels of a 1.0 mm flow cell and cluster amplification was performed. Sequencing-by-synthesis was performed on an Illumina Genome Analyser (GAII). After quality control of the first base incorporation (signal intensities, cluster density) the run was started. All Chip-Seq and input samples were subjected to 36 b single read sequencing run. Processing of the raw data was carried out employing the Illumina 1.2 pipeline version. The data is publicly available at the European Nucleotide Archive (accession number ERP001004).

### Data processing

Public data was downloaded in FASTQ format from the *European Nucleotide Archive*. The details about the different datasets including all accession numbers are summarized in supplementary Table S1. We mapped reads using *Bowtie* (0.12.5) [44] with options −*m* 1 and −*v* 2 which guarantees that only those reads are kept that map uniquely and that contain at most two mismatches when being aligned to the reference, using hg19 and mm9 as reference sequences, see supplementary Table S1 for the number and percentage of mapped reads. Developmental enhancers were obtained from Blow *et al.* [26].

### Peak calling

We run the peak calling software *MACS* (1.4.0) [16] on the resulting BED files using the control data as summarized in supplementary Table S1. We used the *MACS* default parameters, i.e. a p-value cutoff of 10^−5^, except for the tag and effective genome size which had to be adjusted for every experiment and –*mfold* 5,30. As a second step, we solely run *MACS* on every negative control set. Using the resulting control peaks, we “cleaned” the peak lists of the first step by eliminating all treatment peak regions that overlap with these treatment-unspecific control peaks with the help of the tool “intersectBed” from the BEDTools suite [45], the resulting numbers of peaks before and after this “cleaning” procedure are given in supplementary Table S1.

### Bioinformatics analysis

Peaks from the mouse-ChIP-Seq experiments were mapped to the human genome using the UCSC LiftOver tool (−minmatch 0.1). All datasets were iteratively integrated by extending the length of the combined regulatory sites to the span of overlapping peaks. We created a binary matrix that contains for every regulatory site and every factor a ‘1’ if it occurs at that site. Significance of pairwise overlap of genome-wide binding profiles was calculated using a hyper geometric test. Calculated p-values give the probability to observe the number of shared binding events for position-randomized data sets. We sampled from ¼ of the genome-size assuming a minimal overlap of 1 bp to obtain conservative estimates of p-values. Clustering was done on z-score obtained from the hyper geometric tests. Enrichment of histone modifications was calculated on the highest 10% of peaks. All analysis was carried out with R [46], Bioconductor and peakAnalyzer. Supplementary Figure S11 shows mRNA sequencing reads 3000 bp around binding events to demonstrate that proximal binding events are indeed associated with transcription nearby. Supplementary Figure S12 shows the CpG content for different binding events to demonstrate that our observations are not biased toward CpG islands. Combinatorially bound loci in the mouse genome are summarized in supplementary Table S3.

## Supporting Information

Figure S1 Comparison of different cutoffs for peak calling. The diagram shows the percentage of Oct4 bound loci that are bound by Nanog and Sox2. The observed level of co-localization is very similar across data sets with different cutoffs. (**A**) Full data set, all peaks with p<e-05. (B) FDR controlled data set, peaks with p<e-05 and FDR<2%. (**C**) stringent cutoff, the 10% most significant peaks from all peaks with p<e-05.
(TIFF)

Figure S2 Mediator co-localizes with Oct4, Sox2 and Nanog at combinatorially bound enhancers. For every data set, only the 10% most significant peaks of all peaks with p<e-05 are considered. (**A**) Bars indicate the fraction of loci where Med1, Med12 and CTCF binding can be observed, depending on the combination of Oct4, Sox2 and Nanog, indicated by boxes below. Dark boxes indicate binding, white boxes indicate no binding (“AND” relation), light grey boxes with “v” indicate binding of at least one factor (“OR” relation). Both Med1 and Med12 preferentially co-localize at loci bound by Oct4, Sox2 and Nanog simultaneously. CTCF serves as a control to estimate unspecific binding. (**B**) The majority of loci bound by Oct4, Sox2 and Nanog are more than 1000 bp away from the nearest transcription start sites for all possible combinations (indicated by boxes above). Mediator co-localization mainly occurs at distant regulatory sites, showing that the increased overlap of Med1/Med12 at combinatorially bound loci is not caused by promoter specific co-localization.
(TIFF)

Figure S3 Average mouse neuronal progenitor cell H3K27ac ChIP-Seq signal profile around loci bound by Oct4, Sox2 or Nanog in mES cells. Enhancers which are active in mouse development are enriched in H3K27ac in neuronal progenitor cell (red line) supporting that these elements play a role after differentiation of embryonic stem cells.
(TIFF)

Figure S4 The combination of OCT4, SOX2 and NANOG influences conservation of binding events. For every data set, only the 10% most significant peaks of all peaks with p<e-05 are considered. (**A**) Bars indicate the fraction of loci where binding of Nanog, Sox2, Oct4 or CTCF can be observed at the orthologous locus in mouse ES cells for all combinations of OCT4, SOX2 and NANOG in human ES cells as indicated by the boxes below. Dark boxes indicate binding, white boxes indicate no binding (“AND” relation), light grey boxes with “v” indicate binding of at least one factor (“OR” relation). Combinatorial binding of OCT4, SOX2 and NANOG shows the largest fraction of conserved binding for Oct4, Sox2 and Nanog in mouse. (**B**) The fractions of binding combinations in mES cells at conserved loci (for all combinations of binding in human cells as indicated by the boxes above). Combinatorial binding of Oct4, Sox2 and Nanog in mES cells is much higher at combinatorially bound loci in human, suggesting that combinatorial binding is conserved in evolution. (**C**) The fraction of proximal and distant binding sites for conserved and non-conserved binding events, split up according to the combinations of binding as indicated by the boxes above. The majority of conserved binding events are distant regulatory elements.
(TIFF)

Figure S5 Conserved combinatorial binding events are active in development. Bars indicate the fraction of loci which show developmental activity in mouse; boxes below indicate the combination of OCT4, SOX2 and NANOG. 25% of combinatorial binding events which are conserved in mouse and human are active during development.
(TIFF)

Figure S6 The “gene regulatory hotspot” downstream of *SOX21* is functionally conserved between human, mouse and amphioxus. (**A**) Screenshot from the human and mouse genome showing the *SOX21* locus with ChIP-Seq reads for the transcription factors analyzed in this study. (**B**) The human sequence shows reproducible activity in mouse development, picture taken from the VISTA enhancer browser [32] with kind permission from L. Pennacchio. (**C**) The orthologous sequence from amphioxus was tested in zebrafish [33] where it showed reproducible activity in forebrain. Picture in (C) reproduced with kind permission from Genome Research.
(TIFF)

Figure S7 The *ESRRB* locus is bound in a highly species-specific manner. (**A**) Screenshot showing the human *ESRRB* locus. No significant transcription factor binding event can be observed. (**B**) The orthologous locus of *Esrrb* in mouse shows several combinatorial binding events (marked in red). ESRRB might play different roles in human and mouse embryonic stem cells.
(TIFF)

Figure S8 Integrating data from different cell lines identifies functional binding events. (**A**) Shown is the fraction of loci where one, two or three (Oct4, Sox2, Nanog) transcription factor binding events can be observed. Binding events detected in both cell lines are more frequently bound by multiple transcription factors (dotted lines). (**B**) Shown is the fraction of loci where one, two, three or four different factors are binding (OCT4, SOX2, NANOG, p300). The fraction of loci bound by all four factors is much higher when data from embryonic stem cells and embryonal carcinoma cells are combined (dotted lines).
(TIFF)

Figure S9 Binding conservation in embryonic stem cells is increased at developmental enhancers. For every data set, only the 10% most significant peaks of all peaks with p<e-05 are considered. (**A**) Bars indicate the fraction of loci where binding of Nanog, Sox2, Oct4 and CTCF can be observed at the orthologous locus in mouse ES cells for all combinations of OCT4, SOX2 and NANOG in human ES cells discriminated by developmental activity as indicated by the boxes below. Dark boxes indicate “AND” relation, light grey boxes with “v” indicate “OR” relation, “?” indicates no restriction. Combinatorial binding events at developmentally active enhancers show the highest levels of binding conservation between mouse and human ES cells (>40%). (**B**) The fractions of binding combinations in mES cells at conserved loci (for all combinations indicated by the boxes above). The majority of conserved binding events at developmentally active enhancers where OCT4, SOX2 and NANOG bind simultaneously show combinatorial binding of Oct4, Sox2 and Nanog in mouse ES cells. (**C**) The fraction of proximal and distant binding sites for conserved and non-conserved binding events (split up according to the combinations of binding as indicated by the boxes above). The majority of conserved binding events are distant regulatory elements.
(TIFF)

Figure S10 Binding intensities for (**A**) NANOG, (**B**) OCT4 and (**C**) SOX2. Combinatorial binding events show stronger binding intensities than individual binding events, suggesting that the number of false positives, which often show a weak signal, is reduced at combinatorial bound loci.
(TIFF)

Figure S11 mRNA sequencing reads 3000 bp around binding events. Binding events near annotated transcription start sites (red) show higher levels of transcription compared to distant binding events (green). This supports that the majority of binding events is indeed more distant than promoters.
(TIFF)

Figure S12 The majority of binding occurs at low CpG sequences. (**A**) Normalized CpG content for all loci. (**B**) Normalized CpG content separated for proximal and distant binding sites. High CpG sequences mostly occur proximal to the transcription start sites. (**C**) Mediator binding mainly occurs at low CpG sequences. (**D**) Combinatorial bound loci show low CpG content.
(TIFF)

Table S1 Overview of data used in this study.
(XLS)

Table S2 Conserved combinatorially bound loci in mouse and human (hg19).
(CSV)

Table S3 Combinatorially bound loci in mouse (mm9).
(CSV)
