# Supplementary figures and images for: Combinatorial Binding in Human and Mouse Embryonic Stem Cells Identifies Conserved Enhancers Active in Early Embryonic Development
Source: PLoS Comput Biol. 2011 Dec 22;7(12):e1002304. doi: 10.1371/journal.pcbi.1002304 (PMC3245296; doi:10.1371/journal.pcbi.1002304)

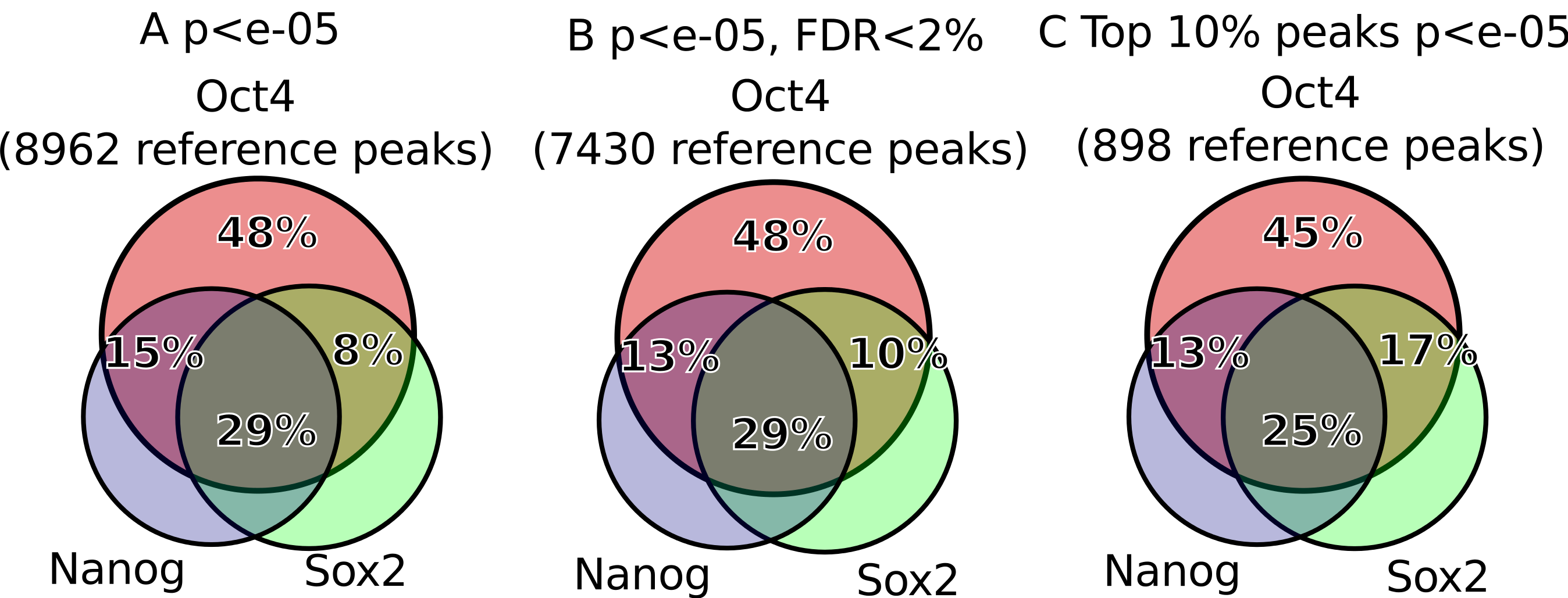

Supplement: Figure S1 — Comparison of different cutoffs for peak calling. The diagram shows the percentage of Oct4 bound loci that are bound by Nanog and Sox2. The observed level of co-localization is very similar across data sets with different cutoffs. (A) Full data set, all peaks with p<e-05. (B) FDR controlled data set, peaks with p<e-05 and FDR<2%. (C) stringent cutoff, the 10% most significant peaks from all peaks with p<e-05. (TIFF) [file pcbi.1002304.s001.tif]

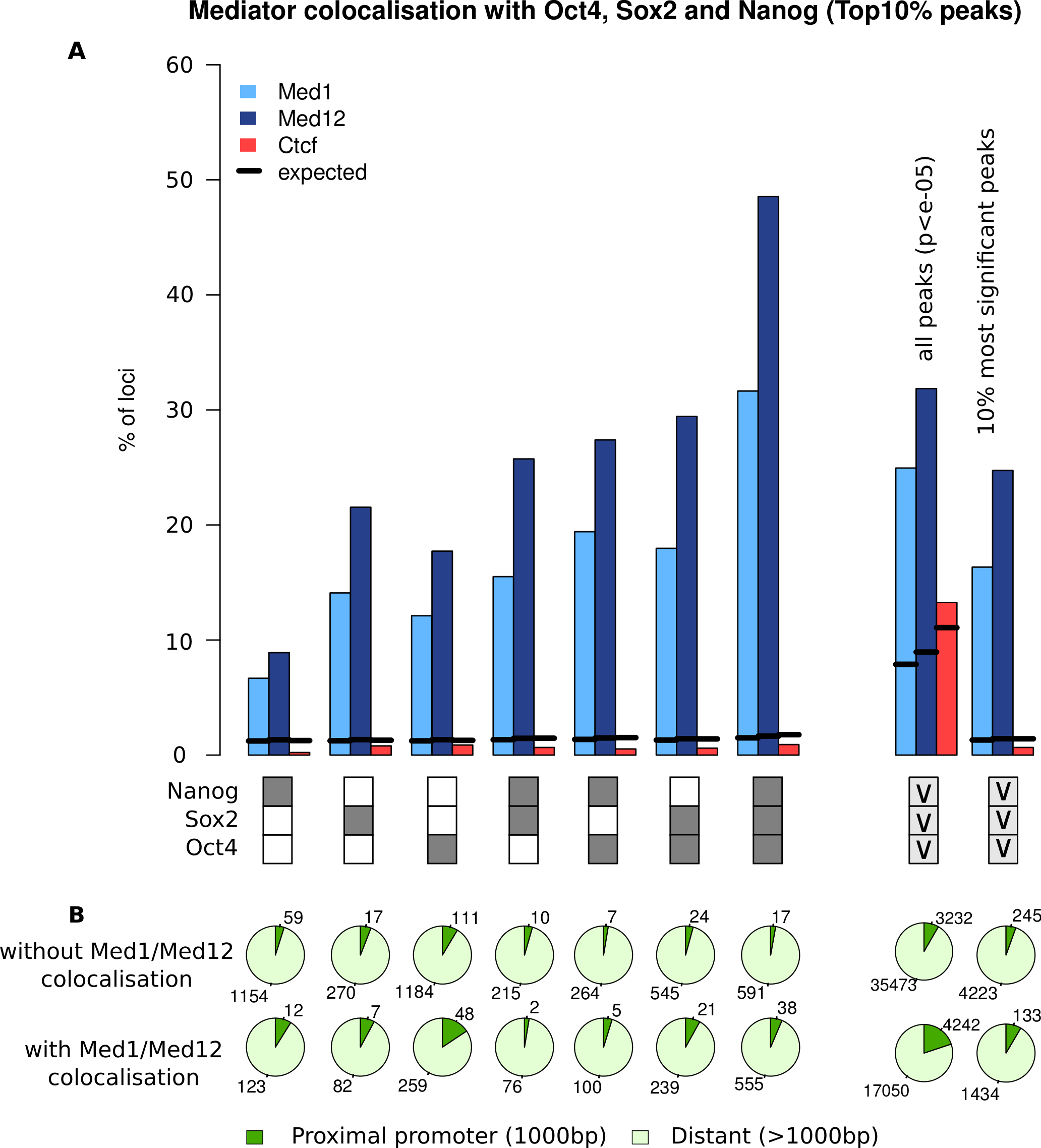

Supplement: Figure S2 — Mediator co-localizes with Oct4, Sox2 and Nanog at combinatorially bound enhancers. For every data set, only the 10% most significant peaks of all peaks with p<e-05 are considered. (A) Bars indicate the fraction of loci where Med1, Med12 and CTCF binding can be observed, depending on the combination of Oct4, Sox2 and Nanog, indicated by boxes below. Dark boxes indicate binding, white boxes indicate no binding (“AND” relation), light grey boxes with “v” indicate binding of at least one factor (“OR” relation). Both Med1 and Med12 preferentially co-localize at loci bound by Oct4, Sox2 and Nanog simultaneously. CTCF serves as a control to estimate unspecific binding. (B) The majority of loci bound by Oct4, Sox2 and Nanog are more than 1000 bp away from the nearest transcription start sites for all possible combinations (indicated by boxes above). Mediator co-localization mainly occurs at distant regulatory sites, showing that the increased overlap of Med1/Med12 at combinatorially bound loci is not caused by promoter specific co-localization. (TIFF) [file pcbi.1002304.s002.tif]

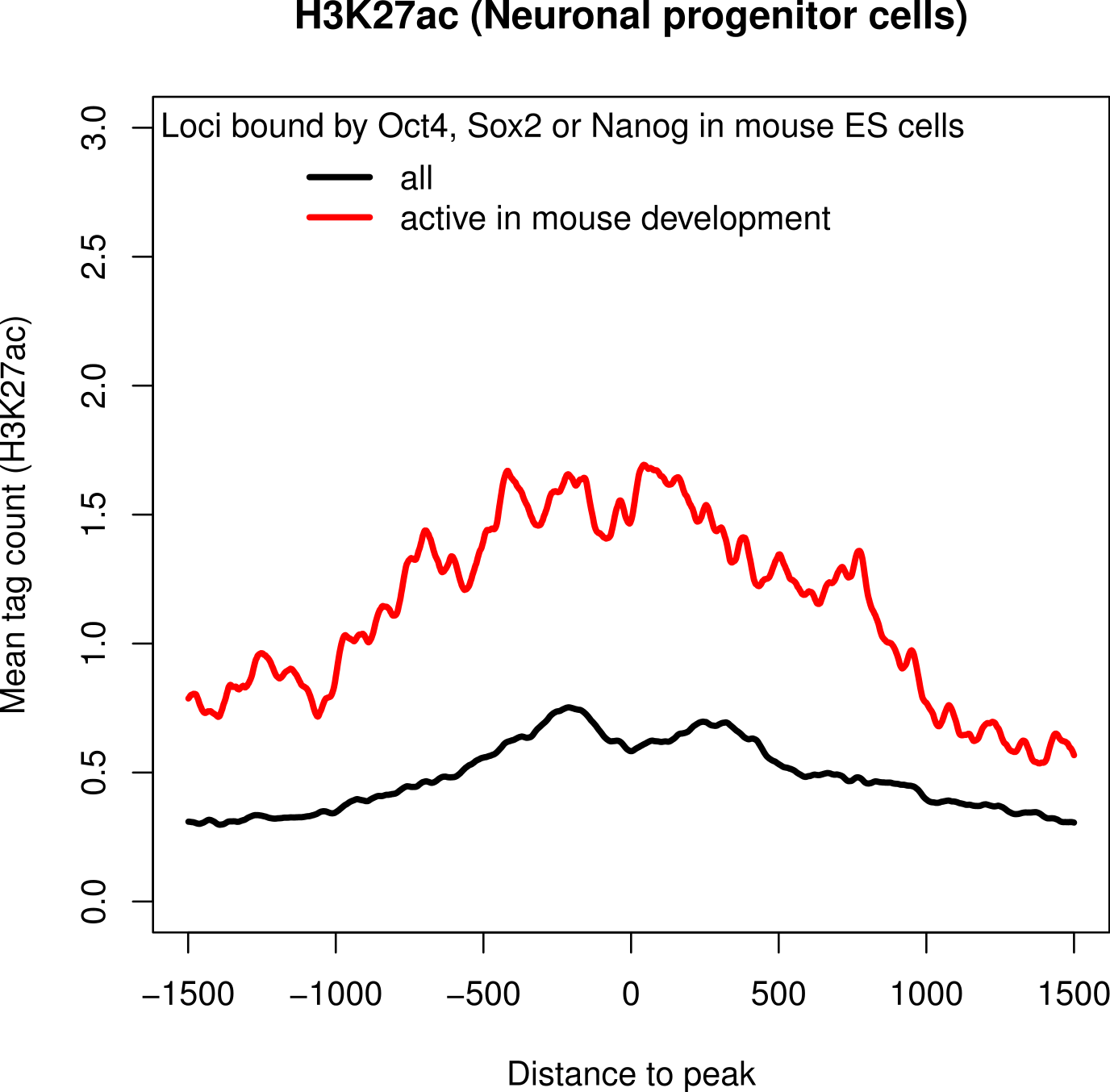

Supplement: Figure S3 — Average mouse neuronal progenitor cell H3K27ac ChIP-Seq signal profile around loci bound by Oct4, Sox2 or Nanog in mES cells. Enhancers which are active in mouse development are enriched in H3K27ac in neuronal progenitor cell (red line) supporting that these elements play a role after differentiation of embryonic stem cells. (TIFF) [file pcbi.1002304.s003.tif]

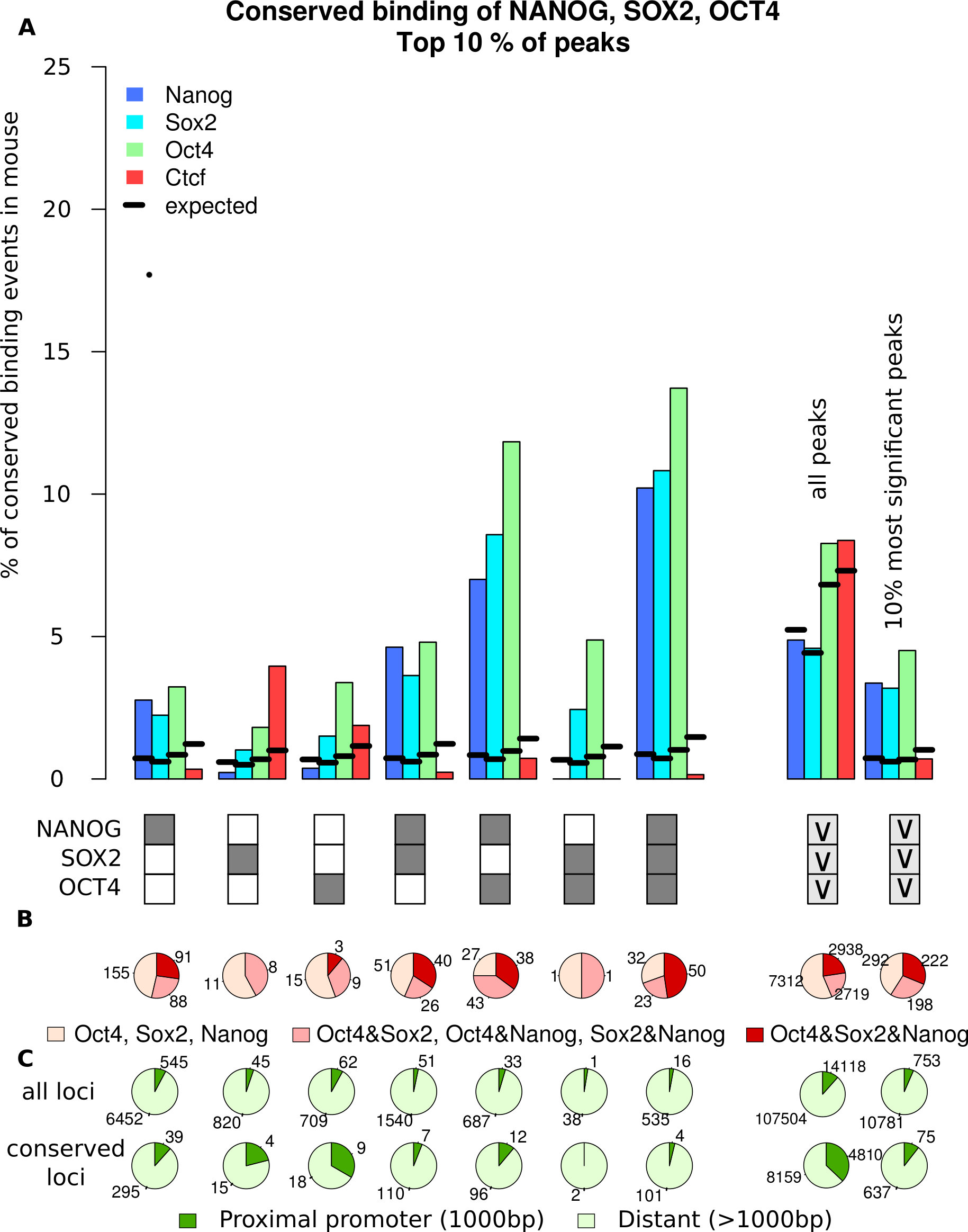

Supplement: Figure S4 — The combination of OCT4, SOX2 and NANOG influences conservation of binding events. For every data set, only the 10% most significant peaks of all peaks with p<e-05 are considered. (A) Bars indicate the fraction of loci where binding of Nanog, Sox2, Oct4 or CTCF can be observed at the orthologous locus in mouse ES cells for all combinations of OCT4, SOX2 and NANOG in human ES cells as indicated by the boxes below. Dark boxes indicate binding, white boxes indicate no binding (“AND” relation), light grey boxes with “v” indicate binding of at least one factor (“OR” relation). Combinatorial binding of OCT4, SOX2 and NANOG shows the largest fraction of conserved binding for Oct4, Sox2 and Nanog in mouse. (B) The fractions of binding combinations in mES cells at conserved loci (for all combinations of binding in human cells as indicated by the boxes above). Combinatorial binding of Oct4, Sox2 and Nanog in mES cells is much higher at combinatorially bound loci in human, suggesting that combinatorial binding is conserved in evolution. (C) The fraction of proximal and distant binding sites for conserved and non-conserved binding events, split up according to the combinations of binding as indicated by the boxes above. The majority of conserved binding events are distant regulatory elements. (TIFF) [file pcbi.1002304.s004.tif]

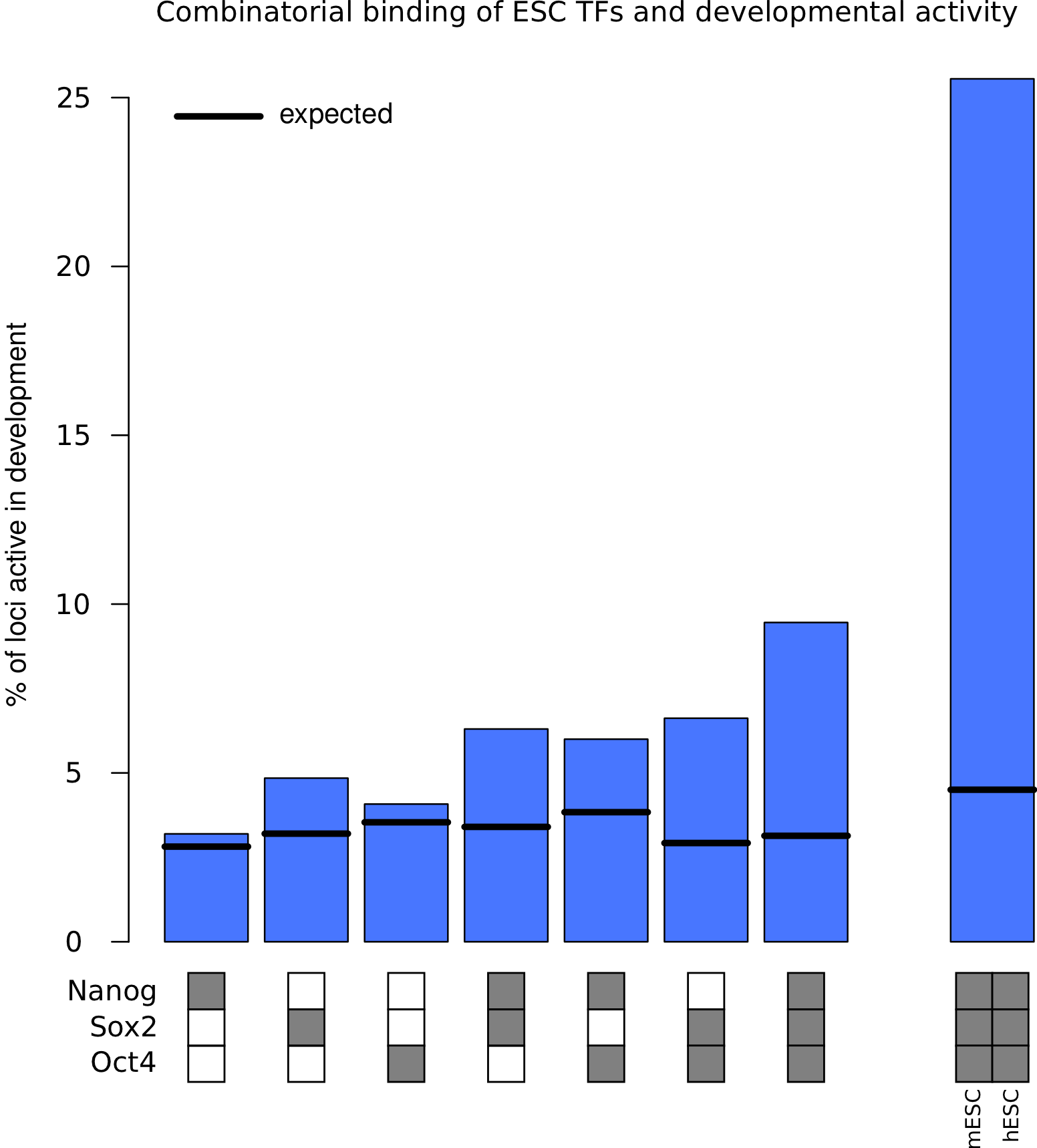

Supplement: Figure S5 — Conserved combinatorial binding events are active in development. Bars indicate the fraction of loci which show developmental activity in mouse; boxes below indicate the combination of OCT4, SOX2 and NANOG. 25% of combinatorial binding events which are conserved in mouse and human are active during development. (TIFF) [file pcbi.1002304.s005.tif]

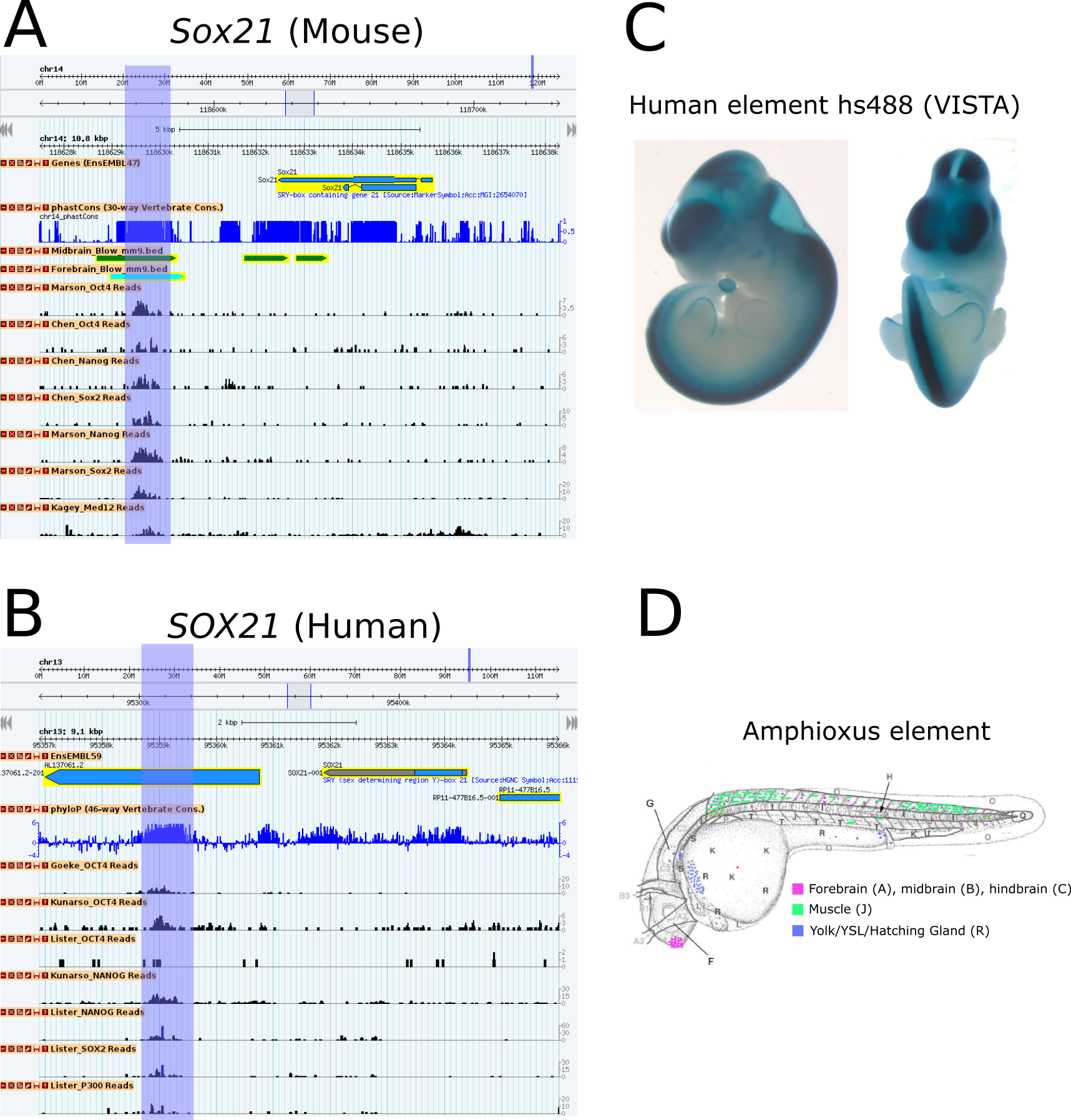

Supplement: Figure S6 — The “gene regulatory hotspot” downstream of SOX21 is functionally conserved between human, mouse and amphioxus. (A) Screenshot from the human and mouse genome showing the SOX21 locus with ChIP-Seq reads for the transcription factors analyzed in this study. (B) The human sequence shows reproducible activity in mouse development, picture taken from the VISTA enhancer browser [32] with kind permission from L. Pennacchio. (C) The orthologous sequence from amphioxus was tested in zebrafish [33] where it showed reproducible activity in forebrain. Picture in (C) reproduced with kind permission from Genome Research. (TIFF) [file pcbi.1002304.s006.tif]

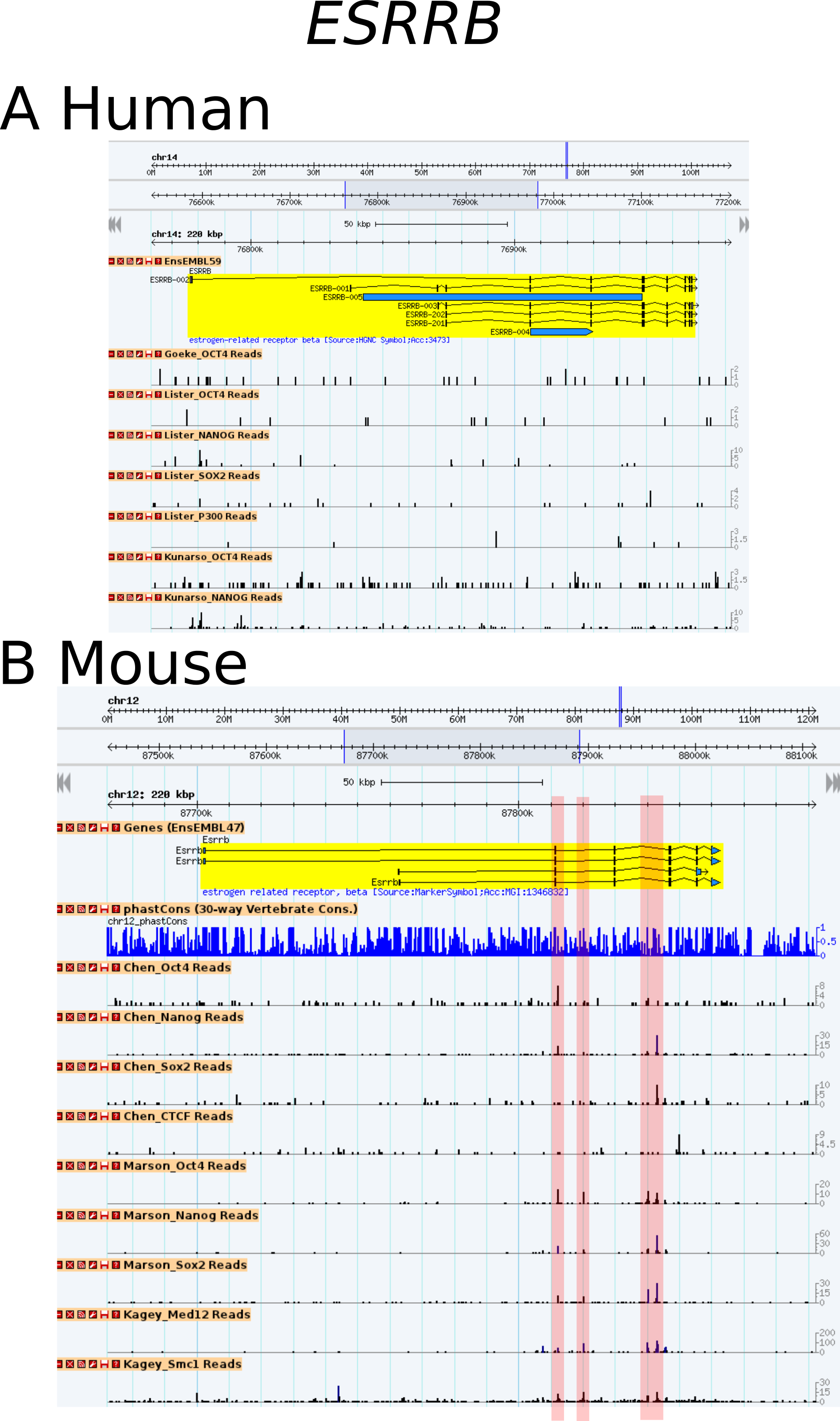

Supplement: Figure S7 — The ESRRB locus is bound in a highly species-specific manner. (A) Screenshot showing the human ESRRB locus. No significant transcription factor binding event can be observed. (B) The orthologous locus of Esrrb in mouse shows several combinatorial binding events (marked in red). ESRRB might play different roles in human and mouse embryonic stem cells. (TIFF) [file pcbi.1002304.s007.tif]

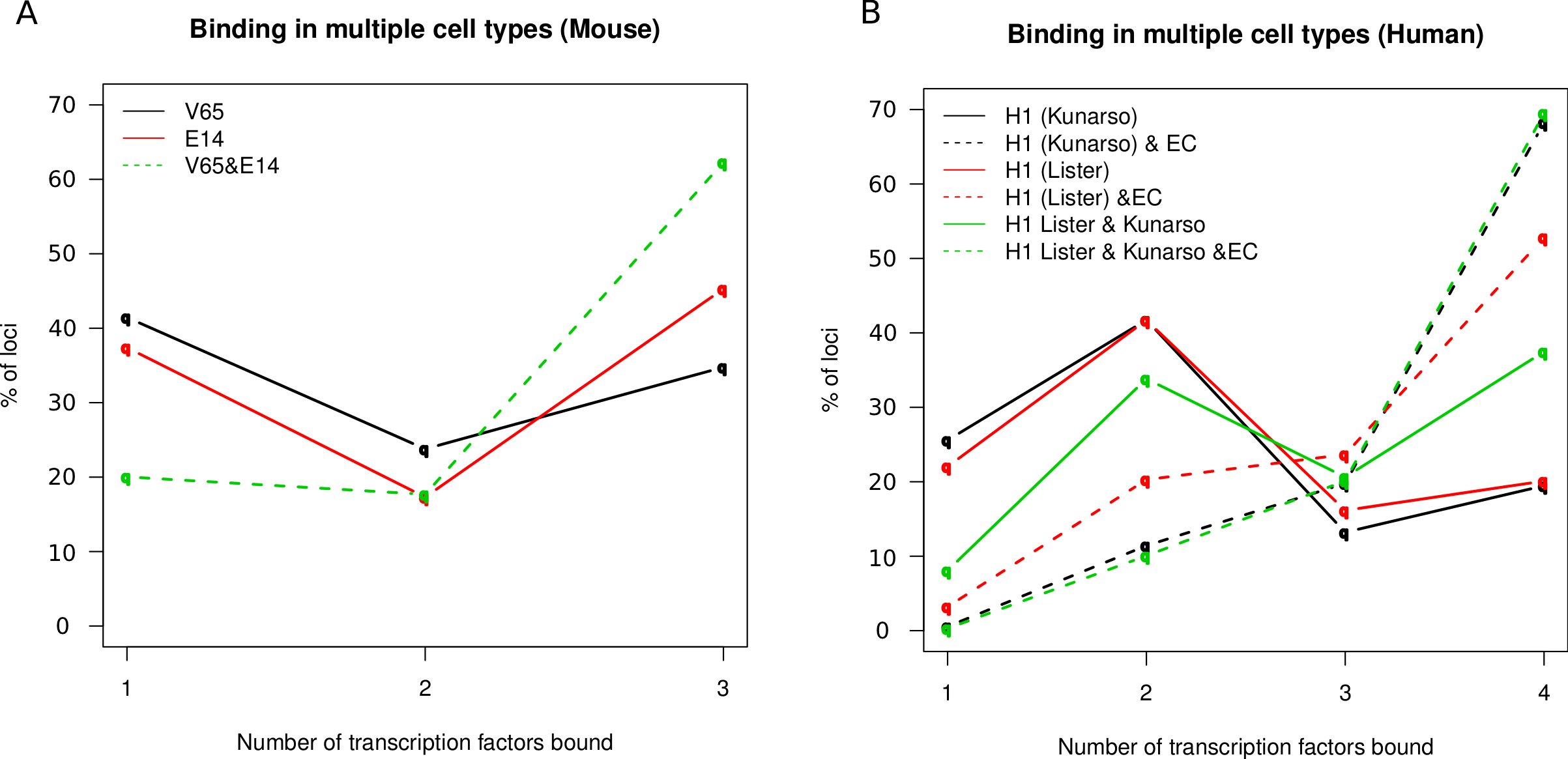

Supplement: Figure S8 — Integrating data from different cell lines identifies functional binding events. (A) Shown is the fraction of loci where one, two or three (Oct4, Sox2, Nanog) transcription factor binding events can be observed. Binding events detected in both cell lines are more frequently bound by multiple transcription factors (dotted lines). (B) Shown is the fraction of loci where one, two, three or four different factors are binding (OCT4, SOX2, NANOG, p300). The fraction of loci bound by all four factors is much higher when data from embryonic stem cells and embryonal carcinoma cells are combined (dotted lines). (TIFF) [file pcbi.1002304.s008.tif]

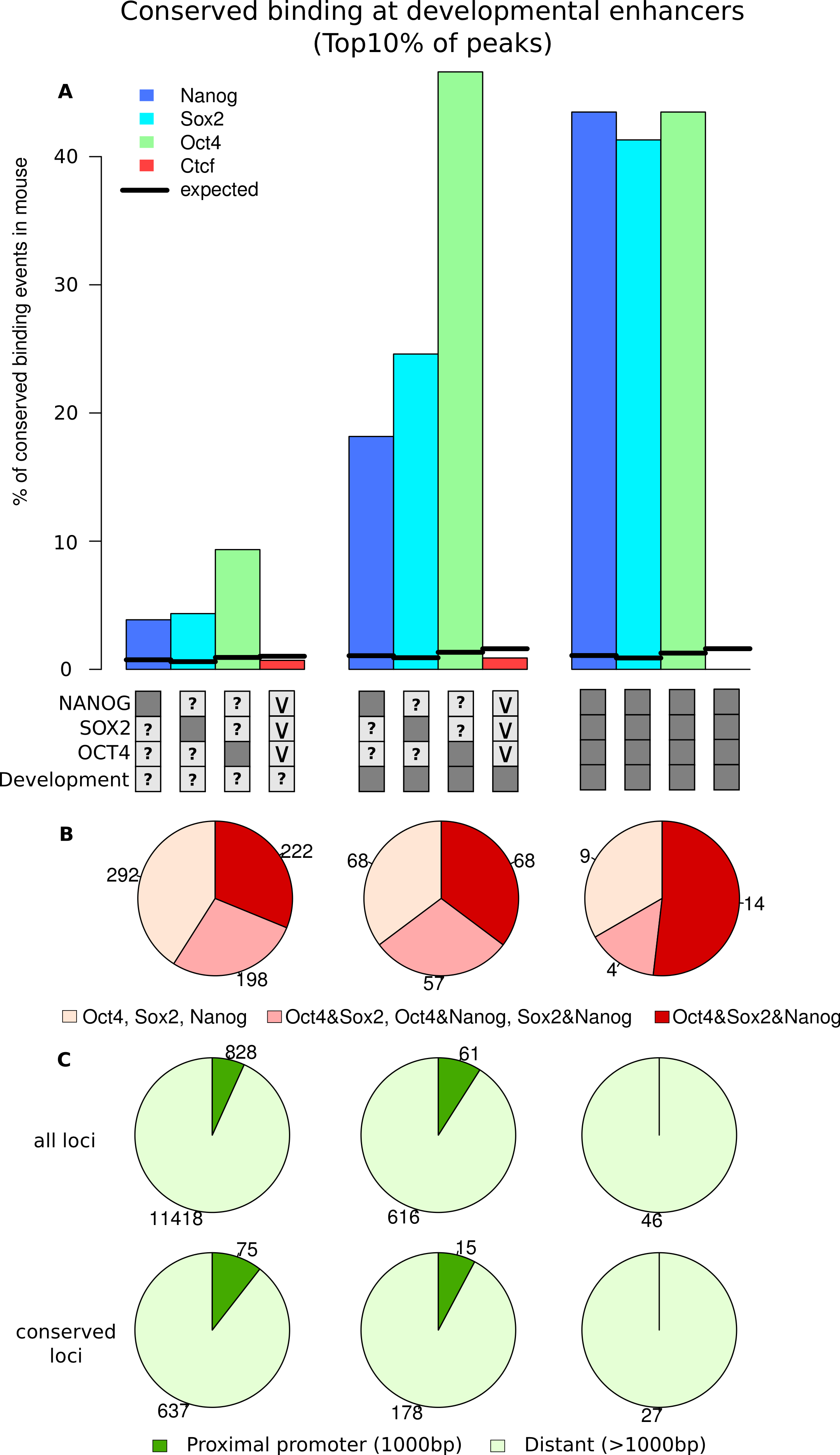

Supplement: Figure S9 — Binding conservation in embryonic stem cells is increased at developmental enhancers. For every data set, only the 10% most significant peaks of all peaks with p<e-05 are considered. (A) Bars indicate the fraction of loci where binding of Nanog, Sox2, Oct4 and CTCF can be observed at the orthologous locus in mouse ES cells for all combinations of OCT4, SOX2 and NANOG in human ES cells discriminated by developmental activity as indicated by the boxes below. Dark boxes indicate “AND” relation, light grey boxes with “v” indicate “OR” relation, “?” indicates no restriction. Combinatorial binding events at developmentally active enhancers show the highest levels of binding conservation between mouse and human ES cells (>40%). (B) The fractions of binding combinations in mES cells at conserved loci (for all combinations indicated by the boxes above). The majority of conserved binding events at developmentally active enhancers where OCT4, SOX2 and NANOG bind simultaneously show combinatorial binding of Oct4, Sox2 and Nanog in mouse ES cells. (C) The fraction of proximal and distant binding sites for conserved and non-conserved binding events (split up according to the combinations of binding as indicated by the boxes above). The majority of conserved binding events are distant regulatory elements. (TIFF) [file pcbi.1002304.s009.tif]

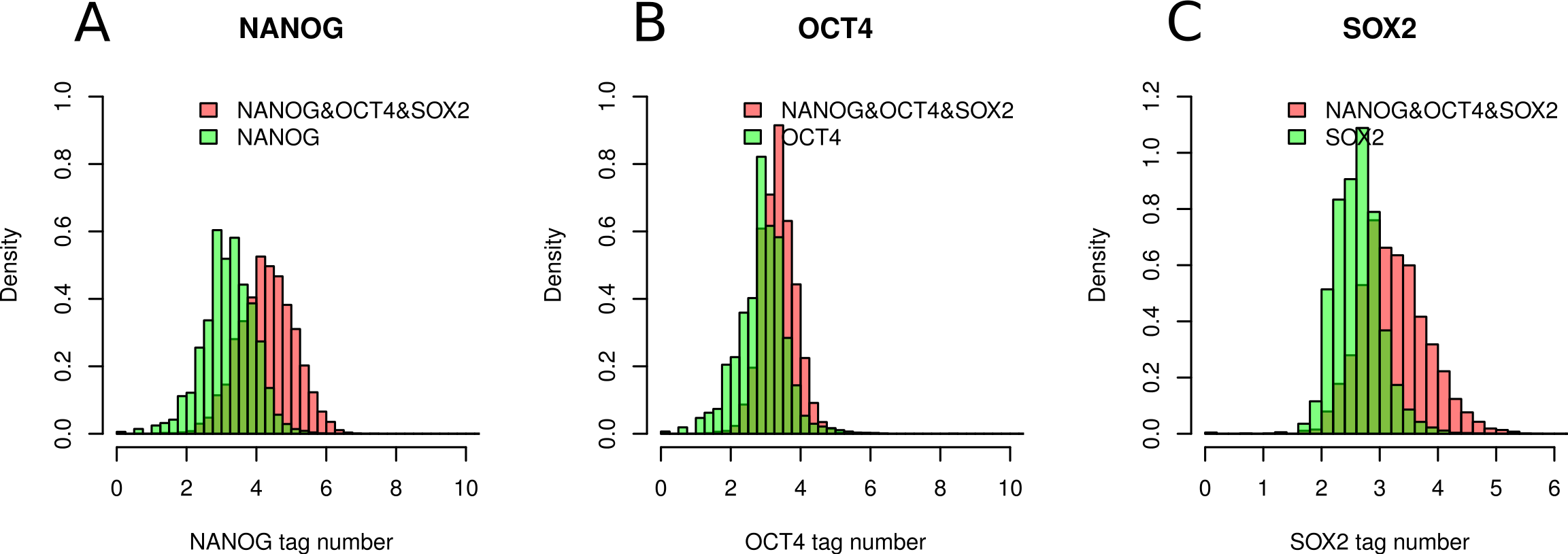

Supplement: Figure S10 — Binding intensities for (A) NANOG, (B) OCT4 and (C) SOX2. Combinatorial binding events show stronger binding intensities than individual binding events, suggesting that the number of false positives, which often show a weak signal, is reduced at combinatorial bound loci. (TIFF) [file pcbi.1002304.s010.tif]

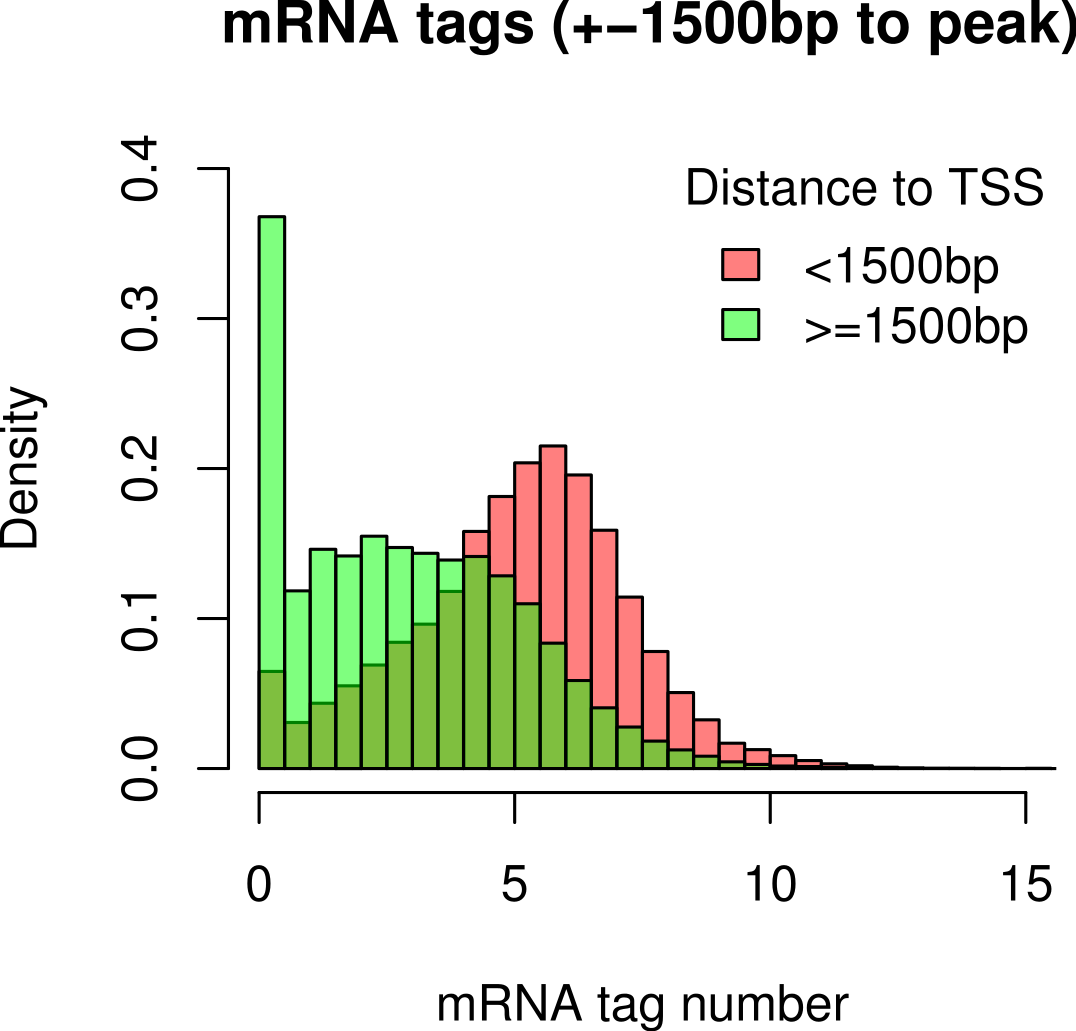

Supplement: Figure S11 — mRNA sequencing reads 3000 bp around binding events. Binding events near annotated transcription start sites (red) show higher levels of transcription compared to distant binding events (green). This supports that the majority of binding events is indeed more distant than promoters. (TIFF) [file pcbi.1002304.s011.tif]

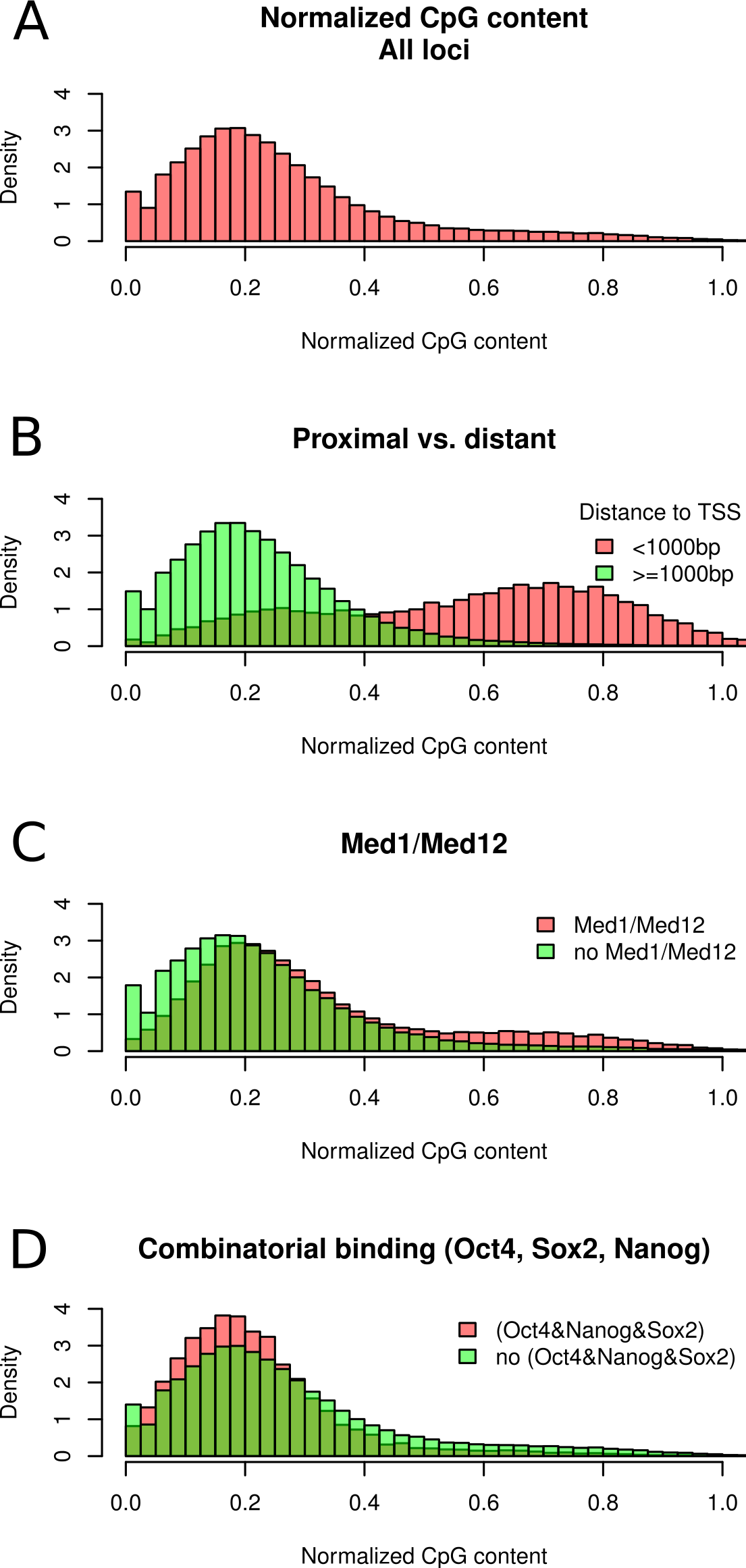

Supplement: Figure S12 — The majority of binding occurs at low CpG sequences. (A) Normalized CpG content for all loci. (B) Normalized CpG content separated for proximal and distant binding sites. High CpG sequences mostly occur proximal to the transcription start sites. (C) Mediator binding mainly occurs at low CpG sequences. (D) Combinatorial bound loci show low CpG content. (TIFF) [file pcbi.1002304.s012.tif]
